# Rapid Methylation of Aryl Bromides Using Air‐Stable DABCO‐Bis(Trimethylaluminum) via Nickel Metallaphotoredox Catalysis

**DOI:** 10.1002/anie.202508710

**Published:** 2025-06-20

**Authors:** Jonas Djossou, Andrea Aloia, Luca Capaldo, Demi D. Snabilié, Morgan Regnier, Jasper H.A. Schuurmans, Antonio Monopoli, Bas de Bruin, Timothy Noël

**Affiliations:** ^1^ Flow Chemistry Group, van ’t Hoff Institute for Molecular Sciences (HIMS) University of Amsterdam Amsterdam 1098 XH The Netherlands; ^2^ Chemistry Department University of Bari Aldo Moro Via Orabona 4 Bari 70125 Italy; ^3^ SynCat Lab, Department of Chemistry, Life Sciences and Environmental Sustainability University of Parma Parco Area delle Scienze 17/A Parma 43124 Italy; ^4^ Homogeneous, Supramolecular and Bio‐inspired Catalysis Group, van ’t Hoff Institute for Molecular Sciences (HIMS) Universiteit van Amsterdam Amsterdam 1098 XH The Netherlands

**Keywords:** DABAl‐Me_3_, Metallaphotoredox, Methylation, Nickel, Organo‐aluminum

## Abstract

We report a metallaphotocatalytic strategy for the selective methylation of (hetero)aryl bromides via nickel‐catalyzed cross‐coupling with bis(trimethylaluminum)‐1,4‐diazabicyclo[2.2.2]octane (DABAl‐Me₃), as a commercially available, air‐stable, and non‐pyrophoric aluminum‐based reagent. The method enables a fast, robust, and scalable methylation protocol that broadly accommodates various functional groups while preventing protodehalogenation. Mechanistic studies confirm the unprecedented generation of methyl radicals from an organo‐aluminum precursor under photoredox conditions, bypassing the limitations of conventional two‐electron pathways. This work expands the toolbox of practical radical precursors and provides a streamlined approach for selective C(sp^2^)─CH_3_ bond formation.

## Graphical abstract

Herein, we report the use of bis(trimethylaluminum)‐1,4‐diazabicyclo[2.2.2]octane (DABAl‐Me_3_) as practical source of radicals for the introduction of valuable methyl groups to aryl bromides via a Nickel/metallaphotocatalytic cross coupling strategy.

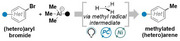

## Introduction

**Scheme 1:**
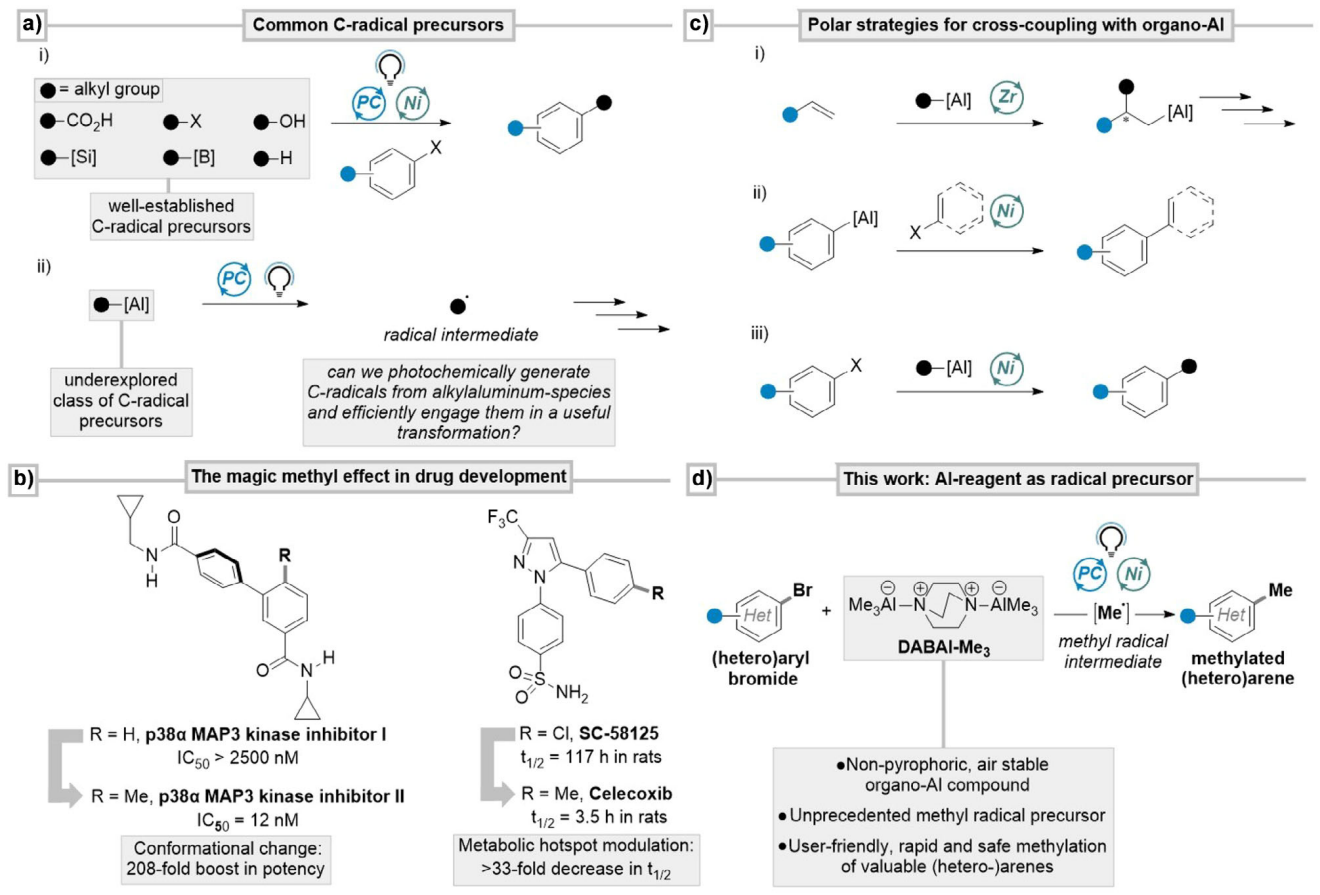
Context, state‐of‐the‐art and this work. a) Common carbon‐based radical precursors used in metallaphotoredox catalysis. b) Examples for the use of organo‐aluminum compounds in two‐electron cross‐coupling methodologies. c) Examples of the magic methyl effect in drug development. d) This work: DABAl‐Me_3_ as unprecedented methyl radical precursor for the methylation aryl bromides under metallaphotoredox conditions.

The growing interest in metallaphotoredox approaches for C(sp^2^)─C(sp^3^) bond formation stems partly from the ability to photochemically generate unstabilized C(sp^3^)‐radicals from readily accessible precursors in a reliable and controlled manner.^[^ ^1^, ^2^, ^3^ ^]^ These systems take advantage of mild reaction conditions, enabling the catalytic generation of nucleophilic alkyl radicals, which can then be selectively coupled with C(sp^2^)‐electrophiles in the presence of earth‐abundant transition metals such as nickel (Scheme 1a i).^[^ ^2^, ^3^, ^4^, ^5^, ^6^, ^7^, ^8^, ^9^, ^10^, ^11^ ^]^

Our group has recently explored the use of trialkylboranes as radical precursors in metallaphotoredox catalysis.^[^ ^12^ ^]^ Given that alkyl boranes can be readily synthesized via the venerable Brown hydroboration, a wide range of primary alkyl fragments could be introduced through light‐mediated B‐alkyl Suzuki–Miyaura coupling. However, incorporating one of the simplest and most ubiquitous alkyl groups—the methyl group—proved challenging. Trimethylborane, a pyrophoric and gaseous reagent, is difficult to obtain and handle safely. While alternative methyl boron sources exist, none have provided a broadly applicable synthetic method for methyl group incorporation in our hands (vide infra). This limitation is of particular significance to medicinal chemistry, where methods for efficient methylation are highly sought after due to the so‐called “magic methyl effect”,^[^ ^13^, ^14^ ^]^ a phenomenon in which strategic methylation can dramatically enhance a molecule's binding affinity, potency, or pharmacokinetics (Scheme 1b).^[^ ^15^ ^]^ In response, a variety of cross‐coupling methodologies were developed to install the methyl group onto aryl rings. While Pd‐catalyzed reactions with methyl nucleophiles can be effective, they frequently require forcing conditions,^[^ ^16^, ^17^, ^18^, ^19^, ^20^, ^21^, ^22^ ^]^ whereas milder protocols employing methyl electrophiles have only recently emerged.^[^ ^23^, ^24^, ^25^ ^]^

These challenges prompted us to explore alternative radical precursors capable of enabling mild and general methylation. In particular, we focused on organoaluminum reagents. Despite being inexpensive, low in toxicity, and the most abundant metal in the Earth's crust,^[^ ^26^ ^]^ aluminum has been largely overlooked as a carbon radical precursor. This raises a fundamental question: can organo‐aluminum compounds generate alkyl radicals under photoredox conditions and participate in synthetically useful cross‐coupling transformations (Scheme 1a ii)? While trimethyl‐aluminum (AlMe₃) is a widely available commodity chemical and organo‐aluminum compounds play a crucial role in large‐scale industrial processes such as the Ziegler–Natta polymerization,^[^ ^27^ ^]^ their application in laboratory settings has been limited by their pyrophoric nature and instability.^[^ ^28^ ^]^ Nevertheless, their lower nucleophilicity combined with pronounced Lewis acidity has enabled the development of a diverse cross‐coupling chemistry (Scheme 1c). These polar strategies include the zirconium‐catalyzed asymmetric carboalumination of alkenes (ZACA), a key method in total synthesis (Scheme 1c i),^[^ ^26^ ^]^ as well as nickel catalyzed cross‐coupling reactions involving unactivated aryl electrophiles with aryl aluminum (Scheme 1c ii)^[^ ^29^, ^30^ ^]^ or alkyl reagents (Scheme 1c iii).^[^ ^31^ ^]^

Motivated by these challenges and recent advances, we were particularly inspired by Doyle's elegant Ni/photocatalytic methylation using acetal‐derived methyl radicals,^[^ ^32^, ^33^ ^]^ as well as Warren's use of bis(trimethylaluminum)‐1,4‐diazabicyclo[2.2.2]octane (DABAl‐Me₃) in Cu‐catalyzed C(sp^3^)–methylation reactions.^[^ ^34^ ^]^ Building on these precedents, we identified DABAl‐Me₃ as a promising candidate for photoredox‐mediated alkyl radical generation (Scheme 1d). DABAl‐Me₃ is a commercially available, air‐stable, and non‐pyrophoric adduct of AlMe₃ with 1,4‐diazabicyclo[2.2.2]octane (DABCO),^[^ ^35^, ^36^ ^]^ exhibiting remarkable stability. This property enables its user‐friendly application as an anionic AlMe₃‐equivalent in organic synthesis.^[^ ^18^, ^34^, ^36^, ^37^ ^]^ Herein, we demonstrate that DABAl‐Me₃ serves as an effective methyl radical source, which can be efficiently captured by a nickel catalyst to achieve the direct methylation of a diverse set of bromo(hetero)arenes (Scheme 1d).

## Results and Discussion

**Scheme 2:**
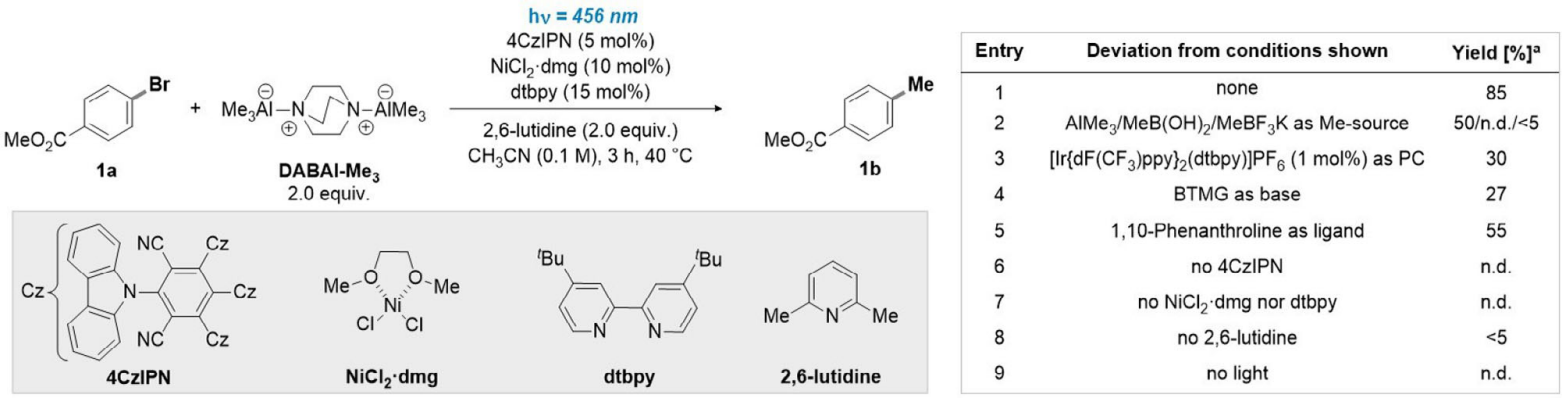
Optimization of the Ni/photocatalytic methylation of 1a with DABAl‐Me_3_. ^a^ Yields determined by ^1^H‐NMR, CH_2_Br_2_ as external standard. 4CzIPN, 2,4,5,6‐tetrakis(9H‐carbazol‐9‐yl)isophthalonitrile; BTMG, 2‐tert‐Butyl‐1,1,3,3‐tetramethylguanidine. n.d., not detected; PC, photocatalyst.

To validate our initial hypothesis, we examined the conversion of aryl bromide **1a** into its corresponding methylated analogue **1b** (Scheme 2). After careful parametric optimization (see Supporting Information), we found that treating **1a** with DABAl‐Me₃ in the presence of 4CzIPN, NiCl₂·dmg, 4,4′‐di‐tert‐butyl‐2,2′‐bipyridyl (dtbpy), and 2,6‐lutidine in CH₃CN, followed by blue light (456 nm) irradiation for 3 h in our standardized UFO photochemical batch reactor,^[^ ^38^ ^]^ yielded the desired compound **1b** in 85% yield (Scheme 2, Entry 1; 65% after isolation). Notably, no reduction side‐products from protodehalogenation could be detected, overcoming a major challenge in the methylation of haloarenes via cross‐coupling.^[^ ^33^ ^]^ Even though AlMe_3_ furnishes **1b** in considerable yield (50%), its practical use is limited due to safety issues, whereas boron‐based methylating reagents were virtually unreactive, likely due to high oxidation potentials (entry 2). Other photocatalysts or bases, such as [Ir{dF(CF_3_)ppy}_2_(dtbpy)]PF_6_ and BTMG, proved to be less effective in the reaction (entries 3, 4). The screening of ligands established *N,N*‐bidentate ligands as optimal (entry 5). Control experiments confirmed the pivotal role of nickel, 2,6‐lutidine, and 4CzIPN (entries 6–8). Notably, no product formation was observed in the absence of light (entry 9), pointing toward a light‐induced reaction mechanism (vide infra).

**Scheme 3:**
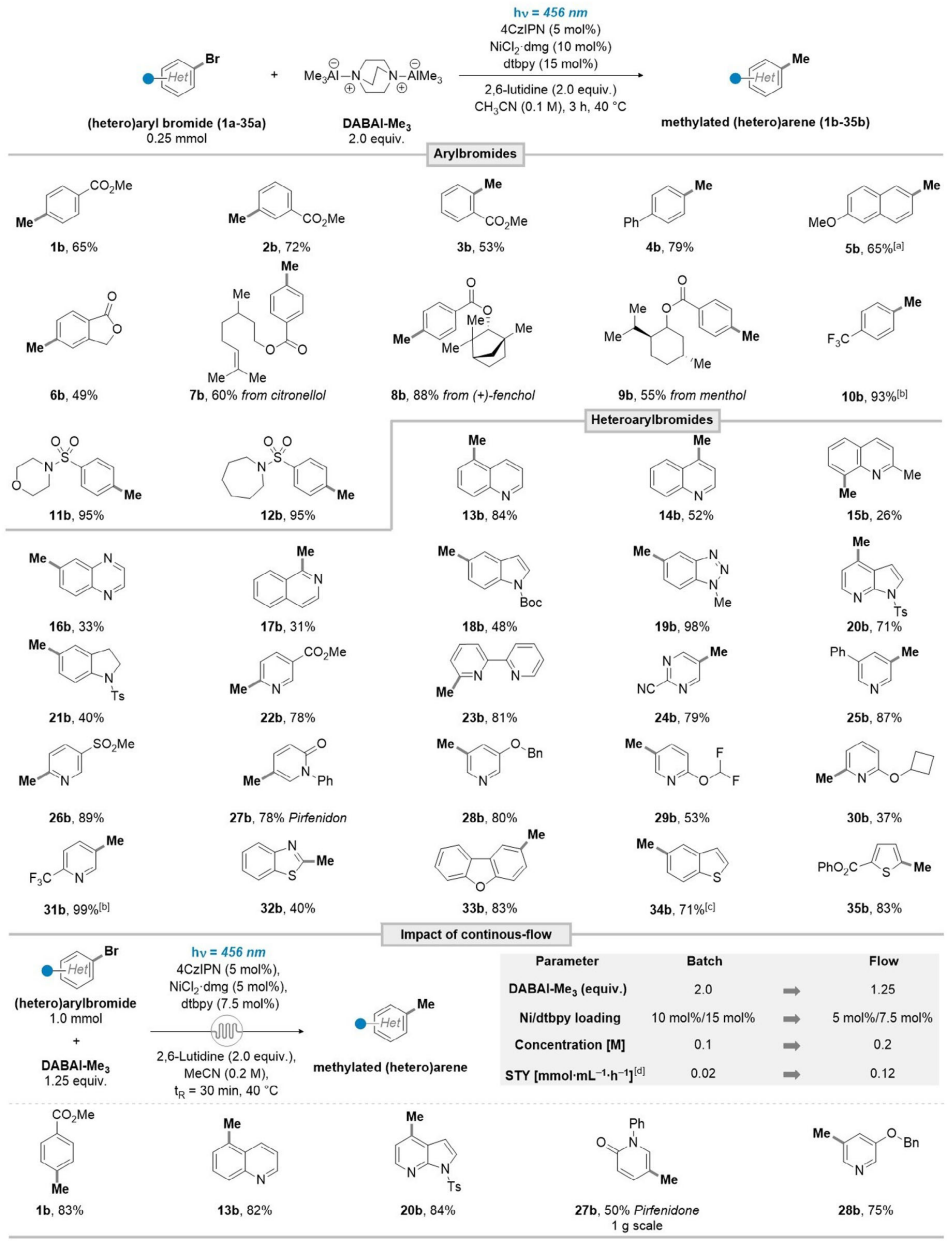
Reaction scope of the nickel metallaphotocatalytic methylation of (hetero)aryl bromides with DABAl‐Me_3_. Conditions for the metallaphotocatalytic methylation of (hetero)aryl bromides with DABAl‐Me_3_: (Hetero)aryl bromide 1a‐35a (0.25 mmol), DABAl‐Me_3_ (2.0 equiv.), 2,6‐lutidine (2.0 equiv.), 4CzIPN (5 mol%), NiCl_2_•dmg (10 mol%), dtbpy (15 mol%), CH_3_CN (0.1 M), N_2_, 456 nm 40 W, 40 °C, 3 h. Continuous‐flow conditions: (Hetero)aryl bromide (1.0 mmol), DABAl‐Me_3_ (1.25 equiv.), 2,6‐lutidine (2.0 equiv.), 4CzIPN (5 mol%), NiCl_2_•dmg (5 mol%), dtbpy (7.5 mol%), CH_3_CN (0.2 M), N_2_, Signify Eagle reactor (λ_max_ = 456 nm, 127 W), 40 °C, t_R_ = 30 min, f_R_ = 0.5 mL min^−1^. ^[a]^ 4–4′‐dimethoxy‐2–2‐bipyridine as ligand. ^[b] 19^F‐NMR‐yield. ^[c]^ 16‐h reaction time. ^[d]^ STY calculated for conversion of 1a into 1b.

Next, we evaluated the generality of our method (Scheme 3). Bromobenzoic esters bearing *o, m, p*‐substituents all underwent smooth methylation, delivering the products in satisfying to good yields (**1b**‐**3b**, 53%–72%). While the phenyl‐substituent furnished the formation of the desired product in good yield (**4b**, 79%), electron‐rich substrates necessitate the use of the more electron‐donating ligand 4,4′‐dimethoxy‐2,2′‐bipyridine (**5b**, 65%). The methylation of structurally more complex benzoyl esters of terpene‐derived alcohols occurred readily (**7b**‐**9b**, 55%–88%). The electron‐withdrawing trifluoromethyl‐substituent facilitated the methylation in excellent yield (**10b**, 93%), whereas the Me‐group could be readily introduced onto aryl sulfonamides decorated with medicinally relevant morpholine and azepan motifs (**11b**, **12b**; 98% respectively). After establishing the methylation of aryl bromides, we sought to expand our methodology towards heteroaryl bromides. Bicyclic systems underwent smooth methylation, as shown for quinolines (**13b**‐**15b**, 26%–84%). Additionally, quinoxaline‐ and isoquinoline‐scaffolds were susceptible to our conditions, albeit furnishing the desired products in reduced yield (**16b**, **17b**; 33%, 31%). Indole‐scaffolds delivered the methylated heteroarenes in moderate to excellent yields (18b‐21b, 48%–98%). The methylation of diversely functionalized pyridines and pyrimidines occurred smoothly (**22b**‐**31b**, 37%–99%). Notably, Pirfenidone **27b**, an FDA‐approved drug for the treatment of idiopathic pulmonary fibrosis,^[^ ^39^ ^]^ could be isolated in 78% yield. Furthermore, we applied our method to challenging oxygen‐ and sulfur‐containing heterocycles, which were efficiently methylated in moderate to good yields (**32b**‐**35b**, 40%–83%). During the exploration of the scope, we encountered diminished yields due to the unexpected volatility of some of the desired products. Additionally, low conversions or decomposition of substrates bearing labile protons or Lewis‐acid sensitive functionalities (e.g., free benzoic acids, aryl pinacolboranes, trityl protecting groups) was found to be a limitation of our methodology (see Supporting Information).

In our effort to facilitate the fast transfer between academic method development and industrial application using enabling technologies, we sought to develop a robust photo‐flow scale‐up procedure.^[^ ^40^ ^]^ A brief readjustment of reaction conditions utilizing a Signify Eagle reactor^[^ ^41^ ^]^ (see Supporting Information for more detail) furnished an improved stoichiometry for DABAl‐Me_3_, decreased Ni‐loading, and a reduced reaction time (see Scheme 3). The improved mass transfer, paired with the higher light intensity resulted in a ∼6‐fold increase in space‐time yield (STY) for compound **1b**. When performed on a 1 mmol scale, entries **1b**, **13b**, **20b**, and **28b** gave comparable or slightly improved yields with respect to the analogous batch reactions, whereas the flow procedure could provide a seamless 24‐fold scale up for the synthesis of **27b** with a good yield on a gram scale.

**Scheme 4:**
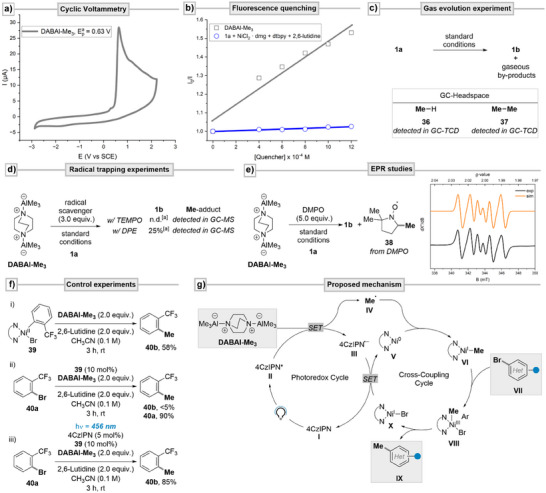
Mechanistic investigation. a) Cyclic voltammetry measurement of DABAl‐Me_3_ at 100 mV S^−1^ in CH_3_CN versus SCE. b) Emission quenching of 4CzIPN* with DABAl‐Me_3_ (grey trace) and the reaction mixture without DABAl‐Me_3_ (blue trace). c) Detection of gaseous by‐products in the batch reaction's headspace via GC‐TCD analysis. d) Radical trapping experiments with TEMPO and DPE under standard conditions. e) EPR‐spectroscopic investigation of the standard reaction in presence of DMPO (black trace) and simulated spectrum (orange trace). f) Control experiments with a nickel oxidative‐addition complex. g) Proposed mechanism for the Ni/photocatalytic methylation of (hetero)aryl bromides with DABAl‐Me_3_ via a radical pathway.

Finally, given the efficacy of our methylation protocol, we were intrigued to gain further insight into the reaction's mechanism, especially regarding the putative involvement of the methyl radical (Scheme 4). First, cyclic voltammetry measurements furnished a non‐reversible oxidation of DABAl‐Me_3_ with an onset at 0.63 V versus SCE, indicating that excited‐state 4CzIPN (E_1/2_(PC*/PC^•–^) = +1.43 V vs. SCE) is a suitable oxidant for the methylating reagent (Scheme 4a).^[^ ^42^ ^]^ After having established the thermodynamic feasibility of DABAl‐Me_3_ reductively quenching excited‐state 4CzIPN, we wondered if it is indeed also kinetically feasible. Consequently, Stern‐Volmer studies were conducted and showed that DABAl‐Me_3_ quenches the fluorescence of 4CzIPN with a higher efficiency compared to the other reaction components (Scheme 4b). These two findings support the assumption of DABAl‐Me_3_ reductively quenching photo‐excited 4CzIPN. In addition, it was experimentally observed that there was a slight pressure build‐up toward reaction completion. This prompted us to perform a GC‐TCD analysis of these gaseous products (Scheme 4c). As expected, methane was detected which could be formed either via protonation of DABAl‐Me_3_^[^ ^36^ ^]^ or H‐atom abstraction of CH_3_CN by the methyl radical.^[^ ^43^ ^]^ Alongside methane, ethane was detected, whose presence could have resulted from Ni‐promoted^[^ ^44^ ^]^ or direct homocoupling^[^ ^45^ ^]^ of methyl radicals. To gain further experimental proof for the presence of methyl radical, trapping experiments were conducted (Scheme 4d). The methylation of **1a** was attempted in the presence of known radical quenchers 2,2,6,6‐tetramethyl‐1‐piperidinyloxy (TEMPO) and 1,1‐diphenylethylene (DPE). In both cases, the respective adducts were detected by GC‐MS, while the product formation was either shut down or vastly suppressed. In addition, EPR measurements were conducted utilizing the spin‐trapping reagent DMPO. The reaction was performed under catalytic conditions with an excess of the spin‐trapping reagent and an aliquot was analyzed by EPR at room temperature (Scheme 4e). The EPR spectrum shows a signal corresponding to the DMPO‐Me persistent radical adduct **38**. The simulated parameters of **38** (orange trace, a*H* = 62.9 MHz, a*N* = 40.3 MHz) correspond well to the values reported in the literature (Scheme 4e).^[^ ^46^ ^]^ Taken together, these experiments provide strong evidence for the efficient formation of methyl radicals under the given photocatalytic conditions. To probe for the possibility of thermal background reactivity in a self‐sustained “dark cycle” promoted by reduction of Ni^II^ by DABAl‐Me_3_,^[^ ^47^ ^]^ we attempted the stoichiometric transmetalation of DABAl‐Me_3_ to the nickel oxidative‐addition complex **39** in the absence of photocatalyst and light (Scheme 4f i). This resulted in the formation of the cross‐coupling product **40b** in 58% yield. However, when employing **39** in catalytic amounts (Scheme 4f ii), only traces of the methylated arene **40b** (<5% yield) could be detected. The majority of the aryl bromide **40a** was left untouched (10% conversion). These observations point to‐wards low‐valent nickel intermediates, generated after a first transmetalation and reductive elimination, which engage in deleterious deactivation pathways rather than engaging in further catalytic reactivity via a two‐electron pathway.^[^ ^48^, ^49^ ^]^ When turning to photocatalytic conditions (Scheme 4f iii), a stark increase in yield was observed (85%). This result is consistent with the high yields obtained during the scope investigation and earlier control experiments, thereby underlining the necessity for both photocatalyst and light. These results taken together, suggest that if a thermal Ni^0^/Ni^II^ or Ni^I^/Ni^III^ catalytic cycle is operative, it is slow compared to the pathways that are enabled under photocatalytic conditions. Based on these experimental observations, we propose a Ni/photocatalytic manifold as the main pathway of product formation, with a plausible mechanism depicted in Scheme 4g. The photocatalyst 4CzIPN **I** is photoexcited to species **II** which swiftly oxidizes DABAl‐Me_3_, thereby generating reduced ground‐state 4CzIPN **III** with concomitant fragmentation of the intermediate radical cation of DABAl‐Me_3_ to the methyl radical **IV** as key intermediate. This alkyl radical species is efficiently trapped by low‐valent nickel species **V**. The thereby generated alkyl‐Ni^I^‐intermediate **VI** oxidatively adds onto (hetero)aryl bromide **VII**. Alternatively, methyl radical trapping and oxidative addition of the aryl bromide could also occur in reverse order. The high‐valent nickel aryl‐alkyl complex **VIII** can efficiently undergo reductive elimination, furnishing the desired methylated arene **IX** and Ni^I^‐intermediate **X**. In a second SET‐event, the nickel complex **X** is reduced by the photocatalyst radical anion **III**, thereby closing both catalytic cycles.

## Conclusion

We have developed a metallaphotocatalytic approach for the selective methylation of (hetero)aryl bromides via Ni‐catalyzed cross‐coupling with DABAl‐Me₃. Compared to previous methods, this strategy leverages a commercially available, air‐stable aluminum‐based reagent and an organophotocatalyst to enable a simplified, fast, and robust procedure that accommodates a broad range of functionalities, delivering clean methylation without protodehalogenation. Furthermore, the advantages of continuous‐flow technology offer opportunities for efficient scale‐up with improved process metrics. Our mechanistic investigation confirms the unprecedented generation of a methyl radical from an organo‐aluminum compound under photoredox conditions. This radical‐based approach bypasses the limitations of conventional two‐electron pathways and expands the synthetic chemist's toolbox of practical radical precursors.

## Supporting Information

The authors have cited additional references within the Supporting Information.^[^ ^46^, ^50^, ^51^, ^52^, ^53^, ^54^, ^55^, ^56^, ^57^, ^58^, ^59^, ^60^, ^61^, ^62^, ^63^, ^64^, ^65^, ^66^, ^67^, ^68^, ^69^, ^70^, ^71^, ^72^, ^73^, ^74^, ^75^, ^76^, ^77^, ^78^, ^79^ ^]^

## Conflict of Interests

The authors declare no conflict of interest.

## Supporting information



Supporting Information

## Data Availability

The data that support the findings of this study are available in Supporting Information of this article.
