# Supplementary material for: Rapid Methylation of Aryl Bromides Using Air‐Stable DABCO‐Bis(Trimethylaluminum) via Nickel Metallaphotoredox Catalysis
Source: Angew Chem Int Ed Engl. 2025 Jun 20;64(33):e202508710. doi: 10.1002/anie.202508710 (PMC12338386; doi:10.1002/anie.202508710)
Supplement: Supplementary file 1 — Supporting Information [file ANIE-64-e202508710-s001.docx]

Supporting Information

Rapid Methylation of Aryl Bromides Using Air-Stable DABCO-*Bis*(Trimethylaluminum) via Nickel Metallaphotoredox Catalysis

Jonas Djossou,^[a]^ Andrea Aloia,^[a,b]^ Luca Capaldo,^[c]^ Demi Snabilié,^[d]^ Morgan Regnier, ^[a]^ Jasper, H.A. Schuurmans,^[a]^ Antonio Monopoli,^[b]^ Bas de Bruin^[d]^ and Timothy Noël*^[a]^

[a]	Jonas Djossou, Dr. Andrea Aloia, Morgan Regnier, Jasper H.A. Schuurmans, Prof. Dr. ing. Timothy Noël
Flow Chemistry Group, van ’t Hoff Institute for Molecular Sciences, University of Amsterdam,1098 XH Amsterdam, The Netherlands.
E-mail: t.noel@uva.nl

[b]	Dr. Andrea Aloia, Prof. Dr. Antonio Monopoli
Chemistry Department, University of Bari Aldo Moro, Via Orabona, 4, 70125 Bari, Italy

[c]	Prof. Dr. Luca Capaldo

SynCat Lab, Department of Chemistry, Life Sciences and Environmental Sustainability, University of Parma, Parco Area delle Scienze 17/A, 43124 Parma, Italy.

[d]	Demi Snabilié, Prof. Dr. Bas de Bruin

Homogeneous, Supramolecular and Bio-inspired Catalysis Group, van ’t Hoff Institute for Molecular Sciences (HIMS), Universiteit van Amsterdam, 1098 XH Amsterdam, The Netherlands.

[1.	General information	3](#_Toc200022880)

[2.	Reactor design	5](#_Toc200022881)

[2.1 Batch reactor (UFO reactor)	5](#_Toc200022882)

[2.2 Flow reactor (Signify Eagle)	6](#_Toc200022883)

[3.	Charts of starting materials	8](#_Toc200022884)

[4. Optimization of reaction conditions	12](#_Toc200022885)

[4.1 Optimization of conditions for reaction with 4-Methyl bromo benzoate (**1a**) and DABAl-Me_3_	12](#_Toc200022886)

[4.2 Adjustment of the reaction conditions for continuous-flow	13](#_Toc200022887)

[4.3 Ethylation experiment with in-situ generated DABCO·AlEt₃	14](#_Toc200022888)

[5. Mechanistic investigations	16](#_Toc200022889)

[5.1 Cyclic voltammetry	16](#_Toc200022890)

[5.2 Stern-Volmer quenching studies	17](#_Toc200022891)

[5.3 GC-headspace analysis	18](#_Toc200022892)

[5.4 Radical trapping	20](#_Toc200022893)

[5.5 EPR studies	22](#_Toc200022894)

[5.6 Control experiments with the Nickel oxidative-addition complex	24](#_Toc200022895)

[6. General procedures (GPs) for preparative experiments	28](#_Toc200022896)

[6.1 GP1: batch conditions with (hetero)aryl bromides	28](#_Toc200022897)

[6.2 GP2: continuous flow conditions	29](#_Toc200022898)

[7. Characterization data	30](#_Toc200022899)

[8. References	46](#_Toc200022900)

[9. NMR spectra	48](#_Toc200022901)

# General information

**Reagents and consumables.** All reagents and solvents were bought from Sigma Aldrich, TCI, Fluorochem, VWR International and Biosolv, and BLD-Pharmatech and used as received. Disposable syringes were purchased from Laboratory Glass Specialist. Syringe pumps were purchased from Chemix Inc. model Fusion 200 Touch. All capillary tubing, microfluidic fittings were purchased from IDEX Health & Science. Product isolation was performed manually, using silica (P60, SILICYCLE), or automatically, by a Biotage® Isolation Four, with Biotage® SNAP KP-Sil 35 g flash chromatography cartridges. TLC analysis was performed using Silica on aluminum foils TLC plates (F254, SILICYCLE) with visualization under ultraviolet light (254 nm and 365 nm) or appropriate potassium permanganate for TLC staining.

**NMR spectroscopy.** ^1^H (400 MHz or 300 MHz), ^13^C (101 MHz or 75 MHz), ^19^F (282 MHz) spectra were recorded unless stated otherwise on ambient temperature using a Bruker AV400 or a Bruker AV300. ^1^H NMR spectra are reported in parts per million (ppm) downfield relative to CDCl_3_ (7.26 ppm); ^13^C NMR spectra are reported in ppm relative to CDCl_3_ (77.2 ppm); ^19^F NMR spectra are referenced to 1-fluoronaphthalene (-124.0 ppm). The multiplicities of signals are designated by the following abbreviations: s (singlet), d (doublet), t (triplet), q (quartet), m (multiplet), dd (doublet of doublets), dt (doublet of triplets), td (triplet of doublets), tt (triplets of triplets), ddd (doublet of doublet of doublets), qd (quartet of doublet). Coupling constants (*J*) are reported in hertz (Hz). NMR data was processed using the MestReNova 14 software package. Known products were characterized by comparing to the corresponding ^1^H NMR, ^13^C NMR, and ^19^F NMR with those available in the literature.

**Melting point.** Melting points were measured using a Büchi Melting Point M-565 apparatus. **Mass spectrometry.** High resolution mass spectra (HRMS) were collected on an AccuTOF LC, JMS-T100LP Mass spectrometer (JEOL, Japan).

**EPR-spectroscopy.** EPR spectra were recorded on a Bruker EMX X-band spectrometer equipped with an ER 4112HV-CF100 He cryostat. CH_3_CN and *^n^*BuCN were pre-dried over oven-dried basic alumina and degassed prior to use.

**UV-Vis spectroscopy.** UV-Vis spectra were recorded with a single-beam Duetta ExSpec equipped with a Xe arc lamp (250-1000 nm) and a CCD camera as a detector. Measurements were performed in a quartz cuvette (optical path: 1 cm). For quantum yield measurements, a double beam spectrophotometer Shimadzu UV2700 equipped with a deuterium lamp (190-350 nm), a halogen lamp (330-900 nm) and a photomultiplier (Hamamatsu R928).

**Cyclic voltammetry.** Cyclic voltammograms (CVs) were recorded using an EmStat3+ (PalmSens BV) potentiostat and a 3-electrodes cell equipped with a glassy carbon disk (3 mm diameter) working electrode, a platinum wire counter electrode and a silver pseudo-reference electrode in a solution of blank electrolyte (0.1 M *^n^*Bu_4_NBF_4_/CH_3_CN) were used. Ferrocene was added at the end of the experiment and a CV recorded to get the potential of the Fc/Fc+ redox couple. The measurements were performed in dry 0.1 M 0.1 M *^n^*Bu_4_NBF_4_/CH_3_CN (5 mL) solutions purged of air with nitrogen. Unless specified, a scan rate of 100 mV·s^-1^ was used.

**Gas-Chromatography**

For analysis a Shimadzu Nexis GC-2030 gas chromatograph, equipped with both a flame ionization detector (FID) and a thermal conductivity detector (TCD), was used. A two-column system (SH-Q-BOND and SH-Msieve 5A) with a bypass was employed to analyze all relevant gaseous compounds. When starting the analysis, 50 µl (sample loop volume) of the gaseous sample is taken and sent to the column. The method uses a split injection (split ratio 5.0) at an injector temperature of 80 °C. A helium carrier gas is used, and all compounds pass the first column (Q-BOND). Next, the light gases are parked in the second column (Msieve 5A), the valve to the second column connected to the TCD (200 °C, helium reference) and FID detector (jetanizer, 280 °C) is opened. The parked gases are flowed to the detectors, during the analysis the oven is kept at 40 °C.

# **Reactor design**

## 2.1 Batch reactor (UFO reactor)

For all batch experiments a homemade, 3D-printed reactor was adopted.^[39]^ The reactor was designed to fit reaction vials and to be equipped with a Kessil lamp PR160L series (λ_em_ = 390 or 456 nm). The reactor was designed in Adobe Inventor 2021 with 4 different parts. The lid (100 mm × 12 mm) is designed to host up to 8 reactions vials and holds the Kessil lamp in the center (A); a fan (SUNON DC Lüfter 24 V; 50x50x15 Vapo RoHS) is mounted on the bottom of the reactor for cooling. The box is designed with holes to allow the air flow to escape the reactor and keep the temperature stable around 40°C (B), as measured by an external thermometer. A reflector is situated underneath the lamp and reflects the photons inside the box to have homogeneous light distribution (C). Finally, the stirring plate adapter (D) was added to fix the system on a stirring plate and provide homogeneous stirring (E). It also spaces the reflector from the plate to ensure continuous air flow from the top to the bottom of the system. All the inside surfaces were covered with reflective tape. An overview of the assembled reactor is shown in figure S1.

**Figure S1**. Overview of the 3D-printed reactor: A) lid designed to host up to 8 reactions vials and hold the Kessil lamp in the center; B) body of the reactor; C) light reflector: it is coated with reflective tape; D) adapter for stirring plate; E) inside of the reactor; G) overall reactor. Copyright: “Photoinduced Halogen-Atom Transfer by N-Heterocyclic Carbene-Ligated Boryl Radicals for C(sp3)–C(sp3) Bond Formation” by our group is licensed under CC BY 4.0.

**Figure S2**. Picture of the assembled reactor equipped with a Kessil lamp (λ_em_ = 456 nm). Copyright: “Photoinduced Halogen-Atom Transfer by N-Heterocyclic Carbene-Ligated Boryl Radicals for C(sp^3^)–C(sp^3^) Bond Formation” by our group is licensed under CC BY 4.0.

## 2.2 Flow reactor (Signify Eagle)

The photochemical prototype reactor (Eagle) was designed and produced by Signify. The reactor consists of six LED light source modules, containing a 456 nm UV-A chip-on-board (COB). Each of these light source modules contain a fan and heat sink combination, which allows the LEDs to be operated efficiently at high power. The Blue LEDs produce monochromatic light with peak wavelength of 450 nm. The six LED modules combined can be operated up to 2.0 A, corresponding to 182 W optical power. The output power of each LED module is individually controllable by a Xitanium LED driver, which works in constant current mode with a maximum current of 2.0 A. The interior of the reactor chamber is cooled with blowers situated on the top cover of the reactor. The LED modules and chamber cooling blowers are connected to a driver box, allowing to set the current of each LED module individually, as well as the cooling blower speed of the reactor chamber. The driver box consists of 6 individual Xitanium LED drivers, which work in Constant Current mode, with a maximum current of 2.0 A. Analog dimming via 1‒10 V control allows for 1‒100% dimming, so minimum current is 20 mA. The individual LED modules can be controlled individually via turning nobs, which set the control voltages of the 1-10 V current circuit. The control voltages are shown in LED displays, in which the value 1.000 corresponds to 10 V. The reactor coil, wrapped around the central unit, is not provided with the reactor, but can be freely chosen by the user. The central unit of the reactor contains a coil with a diameter of 75 mm and a height of 80 mm. The reactor coil is made from PFA capillary and wrapped around the central unit. The assembled reactor is shown in figure S3.

**Figure S3**. Picture of the assembled Signify Eagle reactor.

# Charts of starting materials

The substrates **1a**-**6a**, **10a**-**19a**, **22a**-**29a**, **31a**-**35a** are commercially available. The substrates **9a**,^[50]^ **20a**,^[51]^ **27a**^[52]^ and **29a**^[53]^ were synthesized following literature procedures. Compounds **7a**, **8a**, **21a**, **30a** and **35a** were synthesized as described below.

**Figure S4**. Arylbromide starting materials used in this study.

**3,7-dimethyloct-6-en-1-yl 4-bromobenzoate (7a)**

Citronellol (1.01 mL, 5.5 mmol, 1.1 equiv.) was dissolved in DCM (50 mL) and 4-bromobenzoyl chloride (1.1 g, 5.0 mmol, 1.0 equiv.) and Et_3_N (2.1 mL, 15 mmol, 3.0 equiv.) were added sequentially. The resulting mixture was stirred at room temperature overnight. The reaction mixture was diluted with H_2_O and extracted with EtOAc (3 × 50 mL). The organic layer was dried (Na_2_SO_4_) and concentrated in vacuo. The crude material was purified by flash column chromatography on silica gel (Pentane/EtOAc, 98:2→95:5) to afford the aryl bromide (69%, 1.30 g) as a colorless liquid.

^1^H NMR (300 MHz, CDCl_3_) δ 7.89 (d, *J* = 8.6 Hz, 2H), 7.57 (d, *J* = 8.6 Hz, 2H), 5.15 – 5.03 (m, 1H), 4.42 – 4.26 (m, 2H), 2.12 – 1.89 (m, 2H), 1.89 – 1.74 (m, 1H), 1.72 – 1.49 (m, 8H), 1.48 – 1.32 (m, 1H), 1.32 – 1.15 (m, 1H), 0.96 (d, *J* = 6.4 Hz, 3H).

^13^C {1H} NMR (75 MHz, CDCl_3_) δ 166.1, 131.8, 131.6, 131.2, 129.6, 128.0, 124.6, 63.9, 37.1, 35.6, 29.7, 25.8, 25.5, 19.6, 17.8.

HRMS (FD) m/z calc’d for C_17_H_23_BrO_2_: [M] 338.0881; found: 338.0846.

**(1R,2R,4S)-1,3,3-trimethylbicyclo[2.2.1]heptan-2-yl 4-bromobenzoate (8a)**

(-)-α-Fenchol (848 mg, 5.5 mmol, 1.1 equiv.) was dissolved in DCM (50 mL) and 4-bromobenzoyl chloride (1.1 g, 5.0 mmol, 1.0 equiv.) and Et_3_N (2.1 mL, 15 mmol, 3.0 equiv.) were added sequentially, the resulting mixture was stirred at room temperature overnight. The reaction mixture was diluted with H_2_O and extracted with EtOAc (3 × 50 mL). The organic layer was dried (Na_2_SO_4_) and concentrated in vacuo. The crude material was purified by flash column chromatography on silica gel (Pentane/EtOAc, 98:2→95:5) to afford the aryl bromide (82%, 1.52 g) as a white solid.

^1^H NMR (300 MHz, CDCl_3_) δ 7.92 (d, *J* = 8.6 Hz, 2H), 7.59 (d, *J* = 8.6 Hz, 2H), 4.61 (d, *J* = 1.9 Hz, 1H), 1.97 – 1.84 (m, 1H), 1.83 – 1.72 (m, 2H), 1.71 – 1.62 (m, 1H), 1.60 – 1.46 (m, 1H), 1.30 – 1.20 (m, 2H), 1.18 (s, 3H), 1.10 (s, 3H), 0.83 (s, 3H).

^13^C {1H} NMR (101 MHz, CDCl_3_) δ 166.3, 131.9, 131.2, 129.7, 128.0, 87.2, 48.8, 48.5, 41.6, 40.0, 29.9, 27.0, 26.0, 20.4, 19.6.

HRMS (FD) m/z calc’d for C_17_H_21_Br_1_O_2_: [M] 336.0725; found: 336.0722.

**5-Bromo-1-tosylindoline (21a)**

5-Bromoindoline (337 mg, 1.70 mmol, 1.0 equiv.) was dissolved in DCM (17 mL) and pyridine (0.41 mL, 5.1 mmol, 3.0 equiv.) and p-toluenesulfonyl chloride (357 mg, 1.87 mmol, 1.10 equiv.) were added sequentially. The resulting solution is stirred at room temperature for 16 h, H_2_O is added, the phases are separated, and the aqueous layer is extracted with DCM (3 x). The combined organic layers are washed with 1 M HCl (2 x) and brine, dried over Na_2_SO_4_, filtered and concentrated under reduced pressure. The residue is purified by flash column chromatography on silica gel (Pentane/DCM, 1:1) to afford the aryl bromide as white solid (540 mg, 90%). The spectroscopic data are consistent with the literature.^[54]^

^1^H NMR (300 MHz, CDCl_3_) δ 7.65 (d, *J* = 8.3 Hz, 2H), 7.51 (d, *J* = 8.6 Hz, 1H), 7.34 – 7.15 (m, 4H), 3.90 (t, *J* = 8.5 Hz, 2H), 2.87 (t, *J* = 8.4 Hz, 2H), 2.38 (s, 3H).

^13^C {1H} NMR (75 MHz, CDCl_3_) δ 144.5, 141.4, 134.2, 133.8, 130.8, 129.9, 128.3, 127.4, 116.5, 50.2, 27.8, 21.7.

**2-Bromo-6-cyclobutoxypyridine (30a)**

2-Bromo-6-fluoropyridine (1.0 g, 5.68 mmol, 1.0 equiv.) and cyclobutanol (445 µL, 5.68 mmol, 1.0 equiv.) were dissolved in dry THF (5 mL) and potassium tert-butoxide (1.02 g, 7.39 mmol, 1.30 equiv.) was added at 0°C and then stirred at 0°C for 2 h. Water (20mL) was added into the reaction mixture, and extracted by EA, the organic phase was washed with brine and dried over Na_2_SO_4_. The solvent was removed in vacuo, purification by flash column chromatography on silica gel (Pentane/Et_2_O, 98:2) afforded the aryl bromide as colorless oil (86% yield, 1.12 g).

^1^H NMR (300 MHz, CDCl_3_) δ 7.38 (t, *J* = 7.8 Hz, 1H), 7.02 (d, *J* = 7.5 Hz, 1H), 6.61 (d, *J* = 8.2 Hz, 1H), 5.15 (p, *J* = 7.4 Hz, 1H), 2.53 – 2.38 (m, 2H), 2.21 – 2.03 (m, 2H), 1.90 – 1.60 (m, 2H).

^13^C {1H} NMR (75 MHz, CDCl_3_) δ 162.8, 140.5, 138.9, 120.2, 109.4, 70.6, 30.7, 13.6.

HRMS (FD) m/z calc’d for C_9_H_10_Br_1_N_1_O: [M] 226.9946; found: 226.9953.

**Phenyl 5-bromothiophene-2-carboxylate (35a)**

5-Bromothiophene-2-carboxylic acid (518 mg, 2.5 mmol, 1.0 equiv.) was dissolved in Et_2_O (25 mL) and cooled to 0 °C. Afterwards, phenol (2.5 mmol, 1.0 equiv.), DCC (1 M in DCM, 2.5 mL, 2.5 mmol, 1.0 equiv.) and DMAP (305 mg, 2.5 mmol, 1.0 equiv.) were added sequentially. The reaction mixture was stirred overnight while being allowed to warm to room temperature. The precipitate was collected by filtration and was washed with diethyl ether. The combined filtrates were washed successively with water, 5% aqueous acetic acid, water and brine, dried over Na_2_SO_4_, filtered and concentrated under vacuum. The residue was purified by flash column chromatography on silica gel using (Pentane/EtOAc, 80:20) afforded the aryl bromide as pale-yellow crystals (46% yield, 324 mg).

^1^H NMR (300 MHz, CDCl_3_) δ 7.88 (d, *J* = 1.5 Hz, 1H), 7.55 (d, *J* = 1.5 Hz, 1H), 7.49 – 7.37 (m, 2H), 7.32 – 7.27 (m, 1H), 7.25 – 7.18 (m, 2H).

^13^C {1H} NMR (75 MHz, CDCl_3_) δ 159.5, 150.5, 136.7, 134.0, 130.8, 129.7, 126.4, 121.6, 111.1.

HRMS (EI) m/z calc’d for C_11_H_7_Br_1_O_2_S: [M] 283.9330; found: 283.9338.

# 4. Optimization of reaction conditions

## 4.1 Optimization of conditions for reaction with 4-Methyl bromo benzoate (**1a**) and DABAl-Me_3_

The optimization of the reaction conditions was carried out by studying the methylation of methyl 4-bromobenzoate (**1a**) with DABAl-Me_3_ to give methyl 4-methylbenzoate (**1b**) on a 0.1 mmol scale (vide infra).

In a 7 mL flame dried vial equipped with a stirring bar, **1a** (0.1 mmol), DABAl-Me_3_ (*n* equiv.), base (*n* equiv.), NiCl_2_·dmg (*n* mol%), ligand (*n* mol%), and the chosen photocatalyst (*n* mol%) were dissolved in 1.0 mL of dry CH_3_CN. The mixture was sonicated for 30 seconds to obtain a yellow solution and then bubbled with N_2_ (1 min). The mixture was irradiated for the indicated time with a 40 W Kessil lamp (λ_max_ = 456 nm, full intensity) (see **Table S1**). After irradiation, the solvent was removed, the crude was suspended in EtOAc and filtered through a short silica plug in a Pasteur pipette to remove solids. The solvent was removed, and the sample was analyzed via ^1^H-NMR (CDCl_3_, 7 μL CH_2_Br_2_ as internal standard).

5.1.1 Screening of reaction conditions

**Table S1**. Summary of the investigation of reaction conditions

|  | Entry | Deviation from conditions shown | Yield[%]^a^ |
| --- | --- | --- | --- |
|  | 1 | none | 89 (65)^b^ |
| Methyl source | 2 | AlMe_3_ | 50 |
|  | 3 | MeB(OH)_2_ | n.d |
|  | 4 | MeBF_3_K | <5 |
| DABAl-Me_3_ equiv. | 5 | DABAl-Me_3_ (0.5 equiv.) | 27 |
|  | 6 | DABAl-Me_3_ (1.0 equiv.) | 33 |
|  | 7 | DABAl-Me_3_ (1.5 equiv.) | 61 |
|  | 8 | DABAl-Me_3_ (3.0 equiv.) | 47 |
| Photocatalyst (PC) | 9 | [Ir{dF(CF_3_)ppy}_2_(dtbpy)]PF_6_ (1 mol%) as PC | 30 |
|  | 10 | 4CZTPN as PC | 21 |
|  | 11 | 5CZBN as PC | 26 |
|  | 12 | 3DPA2FBN as PC | <5 |
| Base | 13 | DBU as base | 63 |
|  | 14 | BTMG as base | 27 |
|  | 15 | Cs_2_CO_3_ as base | 29 |
|  | 16 | K_3_PO_4_ as base | 49 |
| Ligand | 17 | 4,4′-Bis(trifluoromethyl)-2,2′-bipyridine | 34 |
|  | 18 | 4,4 -Dimethyl-2,2’-bipyridine | 54 |
|  | 19 | 2,2’-Bipyridine | 11 |
|  | 20 | 1,10-Phenanthroline | 55 |
| Nickel and ligand loading | 21 | NiCl_2_·dmg/dtbpy 1:1 (2 mol%) | 59 |
|  | 22 | NiCl_2_·dmg/dtbpy 1:1 (5 mol%) | 74 |
|  | 23 | NiCl_2_·dmg/dtbpy 1:1 (10 mol%) | 65 |
| Control experiments | 25 | Non-dry, non-degassed CH_3_CN | n.d. |
|  | 26 | No 4CzIPN | n.d. |
|  | 27 | No NiCl_2_·dmg nor dtbpy | n.d. |
|  | 28 | No 2,6-lutidine | <5 |
|  | 29 | No light | n.d. |

DABAl-Me_3_: Bis(trimethylaluminum)-1,4-diazabicyclo[2.2.2]octane; 4CzIPN: 1,2,3,5-tetrakis(carbazol-9-yl)-4,6-dicyanobenzene; 4CZTPN:	2,3,5,6-tetrakis(carbazol-9-yl)-1,4-dicyanobenzene; 5CzBN: 2,3,4,5,6-penta(carbazol-9-yl)benzonitrile; DBU: 1,8-Diazabicyclo(5.4.0)undec-7-ene; BTMG: 2-tert-Butyl-1,1,3,3-tetramethylguanidine; NiCl_2_·dmg: Nickel(II) chloride ethylene glycol dimethyl ether; dtbpy: 4,4′-Di-tert-butyl-2,2′-dipyridyl.

4.2 Adjustment of the reaction conditions for continuous-flow

The optimization of the reaction conditions towards implementation in continuous-flow was carried out by studying the methylation of methyl 4-bromobenzoate (**1a**) with DABAl-Me_3_ to give methyl 4-methylbenzoate (**1b**) on a 0.25 mmol scale (see Table S.2).

A flame dried 20 mL vial was charged with **1a** (0.25 mmol), DABAl-Me_3_ (*n* equiv.), NiCl_2_·dmg (*n* mol%), dtbpy (*n* mol%), 4CzIPN (5 mol%, 13 µmol, 10 mg) and evacuated and backfilled with N_2_. Subsequently, dry 2,6-lutidine (2.0 equiv., 0.5 mmol, 58 µL) and dry CH_3_CN (*n* mL) were added sequentially. The mixture was degassed with N_2_ for 3 min, sonicated for 30 seconds and taken up by a syringe. The syringe was mounted onto a syringe pump and connected to a PFA coil (1.6 mm inner diameter, V = 14.88 mL) which was placed into the Eagle reactor (λ_max_ = 450 nm). The reaction mixture was flowed through the reactor for the indicated residence times at varying light intensity. When the syringe was fully empty, neat CH_3_CN (20 mL) was again loaded into a syringe and injected to collect all product at the end of the reactor in a flask. Afterwards, the solvent was removed, the crude was suspended in EtOAc and filtered through a short silica plug in a Pasteur pipette to remove solids. The solvent was removed, and the sample was analyzed via ^1^H-NMR (CDCl_3_, CH_2_Br_2_ as internal standard). Results are shown in table S2.

**Table S2**. Adjustment of the optimal batch conditions for continuous-flow.

| Entry | Light  intensity [%] | DABAl-Me_3_  equiv. | Concentration [M] | t_R_ [min] | Yield^a^ |
| --- | --- | --- | --- | --- | --- |
| 1 | 100 | 1.5 | 0.1 | 15 | 63 |
| 2 | 100 | 1.5 | 0.2 | 15 | 63 |
| 3 | 100 | 1.25 | 0.2 | 15 | 46 |
| 4 | 50 | 1.25 | 0.2 | 15 | 53 |
| 5 | 50 | 1.25 | 0.2 | 30 | 71 |
| 6 | 70 | 1.25 | 0.2 | 30 | 75 |

4.3 Ethylation experiment with in-situ generated DABCO·AlEt₃

A 7 mL flame dried vial equipped with a stirring bar, was charged with **1a** (54 mg, 0.25 mmol, 1.0 equiv.), DABCO (2.0 equiv., 0.5 mmol, 56 mg), NiCl_2_·dmg (10 mol%, 25 µmol, 5.5 mg), dtbpy (15 mol%, 38 µmol, 10 mg), 4CzIPN (5 mol%, 13 µmol, 10 mg) and the vial was evacuated and backfilled with N_2_. Subsequently, dry 2,6-lutidine (2.0 equiv., 0.5 mmol, 58 µL) and dry CH_3_CN (1.73 mL) were added sequentially. The mixture was degassed with N_2_ for 3 min, and AlEt_3_ (769 µL, 1.3 M in heptane, 1.0 mmol, 4.0 equiv.) was added dropwise under stirring. Upon addition, a white precipitate formed. The septum was sealed with grease and the cap was wrapped in parafilm and the reaction was stirred for 5 min at room temperature. The reaction mixture was sonicated for 30 seconds and irradiated for 3 h with a 40 W Kessil lamp (λ_max_ = 456 nm, full intensity) in the UFO reactor (see figure **S2**). Afterwards, the solvent was removed, the crude was suspended in EtOAc and filtered through a short silica plug in a Pasteur pipette to remove solids. The solvent was removed, and the sample was analyzed via ^1^H-NMR (CDCl_3_, 17.5 µL CH_2_Br_2_ as internal standard) to confirm a yield of **1c** of 87%.

# 5. Mechanistic investigations

5.1 Cyclic voltammetry

The following cyclic voltammograms were recorded at a glassy carbon disk at 100 mV·s^-1^ in a 0.1 M solution of *^n^*Bu_4_NBF_4_.

**Figure S5**. Cyclic voltammetry measurement of the blank solution (green trace), DABCO (orange trace), Ferrocene with DABCO (red trace) and DABAl-Me_3_ (brown trace) at 100 mV/S in CH_3_CN vs SCE.

**Figure S6**. Cyclic voltammetry measurement of DABAl-Me_3_ at 100 mV/S in CH_3_CN vs SCE.

An alternative mechanistic scenario could be elaborated. The dissociation of DABAl-Me_3_ into DABCO and AlMe_3_, would allow free DABCO to serve as sacrificial reductant to the photocatalyst to generate Ni^0^ in situ, which undergoes oxidative addition and polar transmetalation with AlMe_3_ to furnish the desired methylated arene. Beyond a first peak oxidation potential at 0.63 V, DABCO exhibits further oxidation events. In contrast, for DABAl-Me_3_ only a single oxidation peak could be identified, thereby pointing against a pathway for product formation initiated by dissociation of DABAl-Me_3_.

5.2 Stern-Volmer quenching studies

All spectra displayed below were recorded in CH_3_CN (degassed via three cycles of freeze-pump-thaw) in quartz cuvettes (450 – 800 nm; optical path: 1 cm) with the Duetta instrument (see General Information). For sample preparation, under N_2_-atmosphere, a septum-fitted quartz cuvette was charged with a solution of 4CzIPN (3 mL, 10^-5^ M in CH_3_CN). The blank spectrum was recorded at an excitation wavelength of 437 nm with an emission intensity at 560 nm. The stock solutions of the quencher (0.2 M in CH_3_CN) were prepared, containing either all reaction components except DABAl-Me_3_ in the same ratio as in the catalytic batch reaction (e.g. **1a**:2,6-lutidine:NiCl_2_·dmg:dtbpy = 1:2:0.1:0.15; see GP1) or solely DABAl-Me_3_. Afterwards, the appropriate amount of quencher solution was added, the sample was shaken, degassed for 3 min with N_2_, and emission spectra were recorded.

**Figure S7**. UV-Vis emission spectra of 4CzIPN with DABAL-Me_3_ as quencher at each quencher concentration.

**Figure S8**. UV-Vis emission spectra of 4CzIPN with the reaction solution without DABAlMe_3_ as quencher at each quencher concentration.

**Figure S9**. Stern-Volmer plot of the quenching experiments.

To allow a representative comparison of the active quencher species in the catalytic reaction, the quenching behavior the reaction components in their respective ratios and of DABAl-Me_3_ at the same concentration was studied. The reaction solution exhibited slow quenching compared to DABAl-Me_3_. Therefore, it can be concluded that DABAl-Me_3_ is the primary quencher of the excited-state 4CzIPN, thus allowing reduction of high-valent nickel species to occur.

5.3 GC-headspace analysis

A 7 mL flame dried vial equipped with a stirring bar, was charged with **1a** (1.0 equiv., 0.1 mmol, 22 mg), DABAl-Me_3_ (2.0 equiv., 0.2 mmol, 51 mg), NiCl_2_·dmg (10 mol%, 10 µmol, 2.2 mg), dtbpy (15 mol%, 15 µmol, 4.0 mg) and 4CzIPN (5 mol%, 5 µmol, 4.0 mg). The vial is evacuated and backfilled with N_2_. Subsequently, dry 2,6-lutidine (2.0 equiv., 0.2 mmol, 23 µL) and dry CH_3_CN (1.0 mL) were added sequentially. The mixture was degassed with N_2_ for 1 min, the septum was sealed with grease and the cap was wrapped in parafilm. The reaction mixture was sonicated for 30 seconds and irradiated for 3 h with a 40 W Kessil lamp (λ_max_ = 456 nm, full intensity) in the UFO reactor. A sample of the reaction vial’s headspace was taken by syringe and analyzed by GC-TCD.

**Figure S10**. GC-TCD chromatogram of the reaction’s headspace showing methane (7.74 min) and ethane (8.92 min).

At the end of the reaction a slight pressure build-up was observed. The analysis of the gaseous products by GC-TCD analysis allowed to identify these gases as methane and ethane.

5.4 Radical trapping

The study of stoichiometric radical quenching was carried out by study of the reaction of methyl 4-bromobenzoate (**1a**) with DABAl-Me_3_ in presence of 2,2,6,6-tetramethyl-1-piperidinyloxy (TEMPO) and 1,1-diphenylethylene (DPE).

A 7 mL flame dried vial equipped with a stirring bar, was charged with **1a** (1.0 equiv., 0.1 mmol, 22 mg), DABAl-Me_3_ (2.0 equiv., 0.2 mmol, 51 mg), NiCl_2_·dmg (10 mol%, 10 µmol, 2.2 mg), dtbpy (15 mol%, 15 µmol, 4.0 mg) and 4CzIPN (5 mol%, 5 µmol, 4.0 mg). Afterwards, the radical quencher TEMPO (3.0 equiv., 0.3 mmol, 47 mg) or DPE (3.0 equiv., 0.3 mmol, 54 mg) are added respectively and the vial is evacuated and backfilled with N_2_. Subsequently, dry 2,6-lutidine (2.0 equiv., 0.2 mmol, 23 µL) and dry CH_3_CN (1.0 mL) were added sequentially. The mixture was degassed with N_2_ for 1 min, the septum was sealed with grease and the cap was wrapped in parafilm. The reaction mixture was sonicated for 30 seconds and irradiated for 3 h with a 40 W Kessil lamp (λ_max_ = 456 nm, full intensity) in the UFO reactor. The solvent was removed, and the sample was analyzed via ^1^H-NMR (CDCl_3_, 7 μL CH_2_Br_2_ as external standard) and GC-MS.

While DPE suppressed the reactivity (25% ^1^H-NMR), TEMPO (n.d. ^1^H-NMR) completely inhibited product formation. To further gain insight into putative radical trapping adducts, GC-MS analysis was performed which confirmed the presence of each educt in the crude reaction solution.

**Figure S11**. GC-MS spectra of the reaction with TEMPO as radical quencher.

**Figure S12**. GC-MS spectra of the reaction with TEMPO as radical quencher.

5.5 EPR studies

The EPR study was carried out by study of the reaction of methyl 4-bromobenzoate (**1a**) with DABAl-Me_3_ in presence of DMPO and PBN.

Inside a N_2_-filled glove box a 10 mL Schlenk tube is loaded with the spin trap (DMPO: 5.0 equiv., 0.5 mmol, 57 mg; PBN: 5.0 equiv., 0.5 mmol, 89 mg) and transferred outside. The Schlenk flask was charged with **1a** (1.0 equiv., 0.1 mmol, 22 mg), DABAl-Me_3_ (2.0 equiv., 0.2 mmol, 51 mg), NiCl_2_·dmg (10 mol%, 10 µmol, 2.2 mg), dtbpy (15 mol%, 15 µmol, 4.0 mg), 4CzIPN (5 mol%, 10 µmol, 4.0 mg). The vial is evacuated and backfilled with N_2_ and 2,6-lutidine (2.0 equiv., 0.2 mmol, 23 µL) and *^n^*BuCN/ CH_3_CN (5:1, 1.0 mL) were added sequentially. The mixture was degassed with N_2_ for 1 min, the septum was sealed with grease and the cap was wrapped in parafilm. The reaction mixture was sonicated for 30 seconds and irradiated for 2 h with a 40 W Kessil lamp (λ_max_ = 456 nm, full intensity) in the UFO reactor. The reaction mixture is transferred back inside the glovebox, filtered over a syringe filter and transferred to a capillary inside a quartz *J*-Young EPR tube. EPR measurements were recorded at room temperature.

**Figure S13**. Experimental EPR spectrum of the reaction mixture with DMPO measured at room temperature (black) and the simulated EPR spectrum (orange). Settings: 9.646792 MHz, 6.325 mW, modulation amplitude: 4.

**Figure S14**. Experimental EPR spectrum of the reaction mixture with PBN measured at room temperature (black) and the simulated EPR spectrum (red). Settings: 9.64731MHz, 2.000 mW, modulation amplitude: 1

**Table S3**. Simulation parameters of the spin-trapped radical species compared to literature values.

|  | **DMPO simulation**^[55,56]^ | **DMPO literature**^[46]^ | **PBN simulation**^[55,56]^ | **PBN literature**^[46]^ |
| --- | --- | --- | --- | --- |
| *S* | 0.5 |  | 0.5 |  |
| *g*-value | 2.0058 | 2.0054 | 2.0059 | 2.0062 |
| *lw* | 0.598 |  | 0.31 |  |
| a*H* (MHz) | 62.9 | 57.9 | 9.0 | 9.9 |
| *aN* (MHz) | 40.3 | 40.0 | 42.1 | 41.7 |

The presence of the methyl radical intermediate in the reaction solution could be confirmed by spin trapping experiments.

5.6 Control experiments with the Nickel oxidative-addition complex

Inside a N_2_-filled glovebox, a 10 mL *J*-Young flask was charged with a magnetic stir bar and Ni(COD)_2_ (275 mg, 1.0 mmol). Another 10 mL *J*-Young flask was charged with 4,4’-di-tert-butyl-2,2’- dipyridyl (268 mg, 1.0 mmol) and both transferred outside the glovebox. The ligand was dissolved in 5 mL THF (dry, degassed via three cycles of freeze-pump-thaw) and added to the nickel complex dropwise via syringe. Upon completion of the addition, the reaction mixture was stirred room temperature for 1 hour, during which the solution turned purple. Next, 1-bromo-2-(trifluoromethyl)benzene **40a** (4.0 equiv., 4.0 mmol, 545 µL) was added to the reaction mixture dropwise then stirred for 4 hours, resulting in a red solution. Approximately half of the reaction volume was removed by evaporation before the product was precipitated by slow addition of pentane. The product was collected on a fine frit, washed with additional pentane, and thoroughly dried in vacuo to give the desired product **39** as an orange solid (45% yield, 250 mg). The spectroscopic data are consistent with literature values.^[57]^

^1^H NMR (300 MHz, C_6_D_6_) δ 9.64 (s, 1H), 8.64 (d, *J* = 7.6 Hz, 1H), 7.62 (d, *J* = 7.6 Hz, 1H), 7.13 (bs, 4H), 6.98 – 6.89 (m, 1H), 6.53 (bs, 1H), 6.02 (bs, 1H), 0.88 (s, 9H), 0.81 (s, 9H).

^19^F NMR (282 MHz, C_6_D_6_) δ -56.92.

A 7 mL flame dried vial equipped with a stirring bar, was charged with **39** (1.0 equiv., 0.1 mmol, 55 mg) and DABAl-Me_3_ (2.0 equiv., 0.2 mmol, 51 mg). Subsequently, dry 2,6-lutidine (2.0 equiv., 0.2 mmol, 23 µL) and dry CH_3_CN (1.0 mL) were added sequentially, and the vial was wrapped in aluminum foil. The mixture was degassed with N_2_ for 1 min, the septum was sealed with grease and the cap was wrapped in parafilm. The reaction mixture was sonicated for 30 seconds and stirred at room temperature for 3 h. ^19^F-NMR analysis with 1-fluoronaphthalene (15 mg, 0.1 mmol, 1.0 equiv.) as internal standard confirmed a yield of 58% for **40b**.

**Figure S15**. ^19^F-NMR spectrum of the crude reaction of nickel complex **39** with DABAl-Me_3_.

A 7 mL flame dried vial equipped with a stirring bar, was charged with **40a** (1.0 equiv., 0.1 mmol, 23 mg), **39** (10 mol%, 10 μmol, 5.5 mg) and DABAl-Me_3_ (2.0 equiv., 0.2 mmol, 51 mg). Subsequently, dry 2,6-lutidine (2.0 equiv., 0.2 mmol, 23 µL) and dry CH_3_CN (1.0 mL) were added sequentially, and the vial was wrapped in aluminum foil. The mixture was degassed with N_2_ for 1 min, the septum was sealed with grease and the cap was wrapped in parafilm. The reaction mixture was sonicated for 30 seconds and stirred at room temperature for 3 h. ^19^F-NMR analysis with 1-fluoronaphthalene (15 mg, 0.1 mmol, 1.0 equiv.) as internal standard confirmed a yield of <5% for **40b** and 90% remaining **40a**.

**Figure S16**. ^19^F-NMR spectrum of the crude reaction of the reaction of aryl bromide **40a** into **40b** catalyzed by nickel complex **39** in the dark.

A 7 mL flame dried vial equipped with a stirring bar, was charged with **40a** (1.0 equiv., 0.1 mmol, 23 mg), **39** (10 mol%, 10 μmol, 5.5 mg), 4CzIPN (5 mol%, 5 μmol, 4 mg) and DABAl-Me_3_ (2.0 equiv., 0.2 mmol, 51 mg). Subsequently, dry 2,6-lutidine (2.0 equiv., 0.2 mmol, 23 µL) and dry CH_3_CN (1.0 mL) were added sequentially. The mixture was degassed with N_2_ for 1 min, the septum was sealed with grease and the cap was wrapped in parafilm. The reaction mixture was sonicated for 30 seconds and irradiated for 3 h with a 40 W Kessil lamp (λ_max_ = 456 nm, full intensity) in the UFO reactor. ^19^F-NMR analysis with 1-fluoronaphthalene (15 mg, 0.1 mmol, 1.0 equiv.) as internal standard confirmed a yield of 85% for **40b**.

**Figure S17**. ^19^F-NMR spectrum of the crude reaction of the reaction of aryl bromide **40a** into **40b** catalyzed by nickel complex **39** under photocatalytic conditions.

# 6. General procedures (GPs) for preparative experiments

## 6.1 GP1: batch conditions with (hetero)aryl bromides

A 7 mL flame dried vial equipped with a stirring bar, was charged with (hetero)aryl bromide **1a-35a** (0.25 mmol), DABAl-Me_3_ (2.0 equiv., 0.5 mmol, 128 mg), NiCl_2_·dmg (10 mol%, 25 µmol, 5.5 mg), dtbpy (15 mol%, 38 µmol, 10 mg), 4CzIPN (5 mol%, 13 µmol, 10 mg) were added and the vial was evacuated and backfilled with N_2_. Subsequently, dry 2,6-lutidine (2.0 equiv., 0.5 mmol, 58 µL) and dry CH_3_CN (2.5 mL) were added sequentially. The mixture was degassed with N_2_ for 3 min, the septum was sealed with grease and the cap was wrapped in parafilm. The reaction mixture was sonicated for 30 seconds and irradiated for 3 h with a 40 W Kessil lamp (λ_max_ = 456 nm, full intensity) in the UFO reactor (see figure **S2**). After irradiation, the solvent was removed in vacuo and the crude purified by flash column chromatography.

*Notes on purification:* Usually, the cross coupling afforded the methylated (hetero)arenes in moderate to high yields. However, in some cases the separation of the desired product from the corresponding (hetero)aryl bromide proved to be challenging due to the small polarity differences. Moreover, the transformation of (hetero)aryl bromides into their methylated analogues leads to an increase in the volatility of the products, which represents the main cause for decreased isolated yields in some examples. In the case of highly volatile fluorinated examples, ^19^F-NMR spectroscopy was performed by addition of 1-fluoronaphthalene (36.6 mg, 0.25 mmol, 1.0 equiv.) as internal standard to the crude reaction mixture as well as HRMS analysis of an aliquot.

Safety note: Deliberate addition of water to DABAL-Me_3_ causes a strong exothermic reaction and methane liberation. Furthermore, it had been noted that DABAL-Me_3_ samples can ignite on tissue paper especially on “damp” days,^[36]^ but this behavior was not encountered with the samples we commercially obtained. Nonetheless as a precaution, it is recommended to weigh in DABAL-Me_3_ directly into the glass reaction vial.

## 6.2 GP2: continuous flow conditions

A flame dried 20 mL vial was charged with the (hetero)aryl bromide (1.0 mmol), DABAl-Me_3_ (1.25 equiv., 320 mg, 1.25 mmol), NiCl_2_·dmg (5 mol%, 11 mg, 50 µmol), dtbpy (7.5 mol%, 20 mg, 75 µmol), 4CzIPN (5 mol%, 50 µmol, 40 mg) and evacuated and backfilled with N_2_. Subsequently, 2,6-lutidine (2.0 equiv., 2 mmol, 232 µL) and CH_3_CN (5 mL) were added sequentially. The mixture was degassed with N_2_ for 5 min, sonicated for 30 seconds and taken up by a syringe. The syringe was mounted onto a syringe pump and connected to a PFA coil (V = 14.88 mL) which was placed into the Eagle reactor (λ_max_ = 450 nm). The reaction mixture was flowed through the reactor with a flow rate of 0.5 mL/min (30 min residence time) at 70% light intensity (see figure **S3**). When the syringe was fully empty, neat CH_3_CN (20 mL) was again loaded into a syringe and injected to collect all product at the end of the reactor in a flask. Afterwards, the solvent was removed in vacuo and the crude purified by flash column chromatography.

# 7. Characterization data

**Methyl 4-methylbenzoate (1b)**

Prepared according to GP1 from methyl 4-bromobenzoate (54 mg, 0.25 mmol, 1.0 equiv.). Purification by flash column chromatography on silica gel (Pentane/EtOAc, 98:2→95:5) afforded the volatile product as yellow oil (65% yield, 25 mg). The spectroscopic data are in accordance with the literature. ^[58]^

Preparation of **1b** according to GP2 from methyl 4-bromobenzoate (215 mg, 1.0 mmol, 1.0 equiv.) afforded the desired product (83% yield, 125 mg).

^1^H NMR (300 MHz, CDCl_3_) δ 7.93 (d, *J* = 8.2 Hz, 2H), 7.23 (d, *J* = 7.9 Hz, 2H), 3.90 (s, 3H), 2.41 (s, 3H).

^13^C {1H} NMR (75 MHz, CDCl_3_) δ 167.3, 143.7, 129.7, 129.2, 127.6, 52.1, 21.8.

**Methyl 3-methylbenzoate (2b)**

Prepared according to GP1 from methyl 3-bromobenzoate (53.8 mg, 0.25 mmol, 1.0 equiv.). Purification by flash column chromatography on silica gel (Pentane/EtOAc, 95:5) afforded the volatile product as colourless oil (72% yield, 27 mg). The spectroscopic data are in accordance with the literature.^[59]^

^1^H NMR (300 MHz, CDCl_3_) δ 7.90 – 7.79 (m, 2H), 7.42 – 7.28 (m, 2H), 3.91 (s, 3H), 2.40 (s, 3H).

^13^C {1H} NMR (75 MHz, CDCl_3_) δ 167.4, 138.3, 133.8, 130.3, 130.2, 128.4, 126.8, 52.2, 21.4.

**Methyl 2-methylbenzoate (3b)**

Prepared according to GP1 from methyl 3-bromobenzoate (53.8 mg, 0.25 mmol, 1.0 equiv.). Purification by flash column chromatography on silica gel (Heptane/EtOAc, 97:3→95:5) afforded the volatile product as colourless oil (53% yield, 20 mg). The spectroscopic data are in accordance with the literature.^[60]^

^1^H NMR (300 MHz, CDCl_3_) δ 7.95 – 7.86 (m, 1H), 7.45 – 7.34 (m, 1H), 7.30 – 7.18 (m, 2H), 3.89 (s, 3H), 2.60 (s, 3H).

^13^C {1H} NMR (75 MHz, CDCl_3_) δ 168.2, 140.3, 132.1, 131.8, 130.7, 129.7, 125.8, 51.9, 21.8.

**4-methyl-1,1’-biphenyl (4b)**

Prepared according to GP1 from 4-bromo-1,1'-biphenyl (58.3 mg, 0.25 mmol, 1.0 equiv.). Purification by flash column chromatography on silica gel (Pentane) afforded the product as white solid (79% yield, 33 mg). The spectroscopic data are in accordance with the literature.^[61]^

^1^H NMR (300 MHz, CDCl_3_) δ 7.62 (d, *J* = 7.0 Hz, 2H), 7.54 (d, *J* = 8.1 Hz, 2H), 7.46 (t, *J* = 7.5 Hz, 2H), 7.36 (t, *J* = 7.3 Hz, 1H), 7.29 (d, *J* = 7.9 Hz, 2H), 2.44 (s, 3H).

^13^C {1H} NMR (75 MHz, CDCl_3_) δ 141.2, 138.4, 137.1, 129.5, 128.8, 127.1, 127.0, 21.2.

**2-methyl-6-methoxynaphthalene (5b)**

Prepared via a modified GP1 using 4,4’-dimethoxy-2,2’-bipyridine as ligand (8 mg, 38 μmol, 15 mol%) from 2-bromo-6-methoxynaphthalene (59.3 mg, 0.25 mmol, 1.0 equiv.). Purification by flash column chromatography on silica gel (Pentane) afforded the volatile product as white solid (65% yield, 28 mg). The spectroscopic data are in accordance with the literature.^[62]^

^1^H NMR (300 MHz, CDCl_3_) δ 7.66 (dd, *J* = 9.2, 2.5 Hz, 2H), 7.56 (s, 1H), 7.29 (dd, *J* = 8.4, 1.8 Hz, 1H), 7.17 – 7.09 (m, 2H), 3.91 (s, 3H), 2.50 (s, 3H).

^13^C {1H} NMR (101 MHz, CDCl_3_) δ 157.1, 133.1, 132.8, 129.3, 128.8, 128.7, 126.8, 126.70, 118.8, 105.8, 55.4, 21.6.

**5-methylphthalide (6b)**

Prepared according to GP1 from 5-bromophthalide (53.3 mg, 0.25 mmol, 1.0 equiv.). Purification by flash column chromatography on silica gel (Pentane/EtOAc, 85:15→75:25) afforded the volatile product as white solid (49% yield, 26 mg). The spectroscopic data are in accordance with the literature.^[63]^

^1^H NMR (300 MHz, CDCl_3_) δ 7.79 (d, *J* = 7.9 Hz, 1H), 7.33 (d, *J* = 8.1 Hz, 1H), 7.28 (s, 1H), 5.26 (s, 2H), 2.49 (s, 3H).

^13^C NMR (101 MHz, CDCl_3_) δ 171.3, 147.3, 145.4, 130.3, 125.6, 123.3, 122.5, 69.5, 22.2.

**3,7-dimethyloct-6-en-1-yl 4-methylbenzoate (7b)**

Prepared according to GP1 from 3,7-dimethyloct-6-en-1-yl 4-bromobenzoate (85 mg, 0.25 mmol, 1.0 equiv.). Purification by preparative thin-layer chromatography on silica gel (Heptane) afforded the product as colourless oil (60% yield, 41 mg).

^1^H NMR (300 MHz, CDCl_3_) δ 7.93 (d, *J* = 8.2 Hz, 2H), 7.23 (d, *J* = 8.0 Hz, 2H), 5.14 – 5.06 (m, 1H), 4.42 – 4.25 (m, 2H), 2.41 (s, 3H), 2.14 – 1.90 (m, 2H), 1.89 – 1.73 (m, 1H), 1.72 – 1.50 (m, 8H), 1.48 – 1.34 (m, 1H), 1.32 – 1.16 (m, 1H), 0.97 (d, *J* = 6.4 Hz, 3H).

^13^C {1H} NMR (75 MHz, CDCl_3_) δ 166.9, 143.5, 131.5, 129.7, 129.2, 128.0, 124.7, 63.4, 37.1, 35.7, 29.7, 25.8, 25.5, 21.8, 19.6, 17.8.

HRMS (FD) m/z calc’d for C_18_H_26_O_2_: [M] 274.1933; found: 274.1932.

**(1R,2R,4S)-1,3,3-trimethylbicyclo[2.2.1]heptan-2-yl 4-methylbenzoate (8b)**

Prepared according to GP1 from (1R,2R,4S)-1,3,3-trimethylbicyclo[2.2.1]heptan-2-yl 4-bromobenzoate (84 mg, 0.25 mmol, 1.0 equiv.). Purification by preparative thin-layer chromatography on silica gel (Heptane) afforded the product as colourless crystals (88% yield, 60 mg).

^1^H NMR (300 MHz, CDCl_3_) δ 7.96 (d, *J* = 7.9 Hz, 2H), 7.25 (d, *J* = 7.8 Hz, 2H), 4.61 (s, 1H), 2.41 (s, 3H), 2.02 – 1.88 (m, 1H), 1.86 – 1.72 (m, 2H), 1.70 – 1.62 (m, 1H), 1.60 – 1.42 (m, 1H), 1.29 – 1.22 (m, 2H), 1.18 (s, 3H), 1.11 (s, 3H), 0.84 (s, 3H).

^13^C {1H} NMR (75 MHz, CDCl_3_) δ 167.1, 143.5, 129.7, 129.2, 128.1, 86.6, 48.8, 48.6, 41.61, 40.0, 29.9, 27.0, 26.1, 21.8, 20.4, 19.6.

HRMS (FD) m/z calc’d for C_18_H_24_O_2_: [M] 272.1776; found: 272.1768.

**(1R,2S,5R)-5-methyl-2-(propan-2-yl)cyclohexyl 4-methylbenzoate (9b)**

Prepared according to GP1 from (1R,2S,5R)-2-isopropyl-5-methylcyclohexyl 4-bromobenzoate (84.9 mg, 0.25 mmol, 1.0 equiv.). Purification by flash column chromatography on silica gel (Pentane/EtOAc, 100:0→97:3) afforded the product as colorless oil (55% yield, 46.7 mg). The spectroscopic data are in accordance with the literature.^[64]^

^1^H NMR (300 MHz, CDCl_3_) δ 7.94 (d, *J* = 8.2 Hz, 2H), 7.23 (d, *J* = 8.0 Hz, 2H), 4.92 (td, *J* = 10.8, 4.4 Hz, 1H), 2.41 (s, 3H), 2.17 – 2.08 (m, 1H), 2.02 – 1.90 (m, 1H), 1.77 – 1.68 (m, 2H), 1.62 – 1.47 (m, 2H), 1.19 – 1.02 (m, 2H), 0.97 – 0.87 (m, 7H), 0.79 (d, *J* = 6.9 Hz, 3H).

^13^C {1H} NMR (150 MHz, CDCl_3_) δ 166.2, 143.3, 129.6, 129.0, 128.2, 74.6, 47.3, 41.0, 34.4, 31.5, 26.5, 23.7, 22.1, 21.6, 20.8, 16.6.

**4-tosylmorpholine (10b)**

Prepared according to GP1 from 4-[(4-Bromophenyl)sulfonyl]morpholine (77 mg, 0.25 mmol, 1.0 equiv.). Purification by flash column chromatography on silica gel (Pentane/EtOAc, 95:5→90:10) afforded the product as white solid (95% yield, 57 mg). The spectroscopic data are in accordance with the literature.^[65]^

^1^H NMR (300 MHz, CDCl_3_) δ 7.64 (d, *J* = 7.8 Hz, 2H), 7.34 (d, *J* = 7.9 Hz, 2H), 3.78 – 3.69 (m, 2H), 3.03 – 2.94 (m, 2H), 2.45 (s, 3H).

^13^C {1H} NMR (75 MHz, CDCl_3_) δ 144.1, 132.2, 129.9, 128.6, 66.3, 46.1, 21.7.

**4-tosylazepan (11b)**

Prepared according to GP1 from 1-((4-bromophenyl)sulfonyl)azepane (70 mg, 0.25 mmol, 1.0 equiv.). Purification by flash column chromatography on silica gel (Heptane/EtOAc, 85:15) afforded the product as white solid (95% yield, 60 mg). The spectroscopic data are in accordance with the literature.^[66]^

^1^H NMR (300 MHz, CDCl_3_) δ 7.67 (d, *J* = 8.0 Hz, 2H), 7.28 (d, *J* = 8.0 Hz, 2H), 3.30 – 3.20 (m, 4H), 2.41 (s, 2H), 1.76 – 1.66 (m, 4H), 1.64 – 1.53 (m, 4H).

^13^C NMR (75 MHz, CDCl_3_) δ 143.0, 136.8, 129.7, 127.1, 48.3, 29.3, 27.1, 21.6.

**1-methyl-4-(trifluoromethyl)benzene (12b)**

Prepared according to GP1 from 4-trifluoromethylphenyl bromide (56 mg, 0.25 mmol, 1.0 equiv.). After the irradiation, 1-fluoronaphthalene (36.6 mg, 0.25 mmol, 1.0 equiv.) was added as internal standard and ^19^F-NMR analysis was performed, to confirm a yield of 95%. The spectroscopic data are in accordance with the literature.^[67]^

^19^F NMR (282 MHz, CDCl_3_) δ -63.10.

HRMS (EI) m/z calc’d for C_8_H_8_F_3_: [M+H] 161.0578; found: 161.0452.

**5-methylquinoline (13b)**

Prepared according to GP1 from 5-bromoquinoline (52 mg, 0.25 mmol, 1.0 equiv.). Purification by flash column chromatography on silica gel (Pentane/EtOAc, 97.5:2.5) afforded the volatile product as colorless oil (84% yield, 30 mg). The spectroscopic data are in accordance with the literature.^[68]^

Preparation of **1b** according to GP2 from 5-bromoquinoline (208 mg, 1.0 mmol, 1.0 equiv.) afforded the desired product (82% yield, 117 mg).

^1^H NMR (300 MHz, CDCl_3_) δ 8.82 (d, *J* = 1.1 Hz, 1H), 8.22 (d, *J* = 8.5 Hz, 1H), 7.87 (d, *J* = 8.5 Hz, 1H), 7.58 – 7.44 (m, 1H), 7.39 – 7.22 (m, 2H).

^13^C {1H} NMR (75 MHz, CDCl_3_) δ 150.0, 148.6, 134.6, 132.5, 129.2, 127.7, 127.0, 120.7, 18.6.

**4-methylquinoline (14b)**

Prepared according to GP1 from 4-bromoquinoline (52 mg, 0.25 mmol, 1.0 equiv.). Purification by flash column chromatography on silica gel (Pentane/EtOAc, 95:5→90:10) afforded the volatile product as colourless oil (87% yield, 31 mg). The spectroscopic data are in accordance with the literature.^[68]^

^1^H NMR (300 MHz, CDCl_3_) δ 8.77 (d, *J* = 4.4 Hz, 1H), 8.10 (d, *J* = 8.2 Hz, 1H), 7.99 (d, *J* = 8.3 Hz, 1H), 7.74 – 7.65 (m, 1H), 7.59 – 7.51 (m, 1H), 7.22 (d, *J* = 4.2 Hz, 1H), 2.70 (s, 3H).

^13^C {1H} NMR (75 MHz, CDCl_3_) δ 150.3, 148.1, 144.4, 130.1, 129.2, 128.4, 126.4, 123.9, 122.0, 18.8.

**4-methyl-1-tosyl-1H-pyrrolo[2,3-b]pyridine (15b)**

Prepared according to GP1 from 8-bromo-2-methylquinoline (56 mg, 0.25 mmol, 1.0 equiv.). Purification by flash column chromatography on silica gel (Pentane/EtOAc, 95:5→90:10) afforded the volatile product as colourless oil (26% yield, 10 mg). The spectroscopic data are in accordance with the literature.^[69]^

^1^H NMR (300 MHz, CDCl_3_) δ 8.00 (d, *J* = 8.4 Hz, 1H), 7.61 (d, *J* = 9.7 Hz, 1H), 7.53 (d, *J* = 7.0 Hz, 1H), 7.36 (t, *J* = 7.5 Hz, 1H), 7.27 (d, *J* = 8.4 Hz, 1H), 2.81 (s, 2H), 2.76 (s, 3H).

^13^C {1H} NMR (75 MHz, CDCl_3_) δ 158.0, 147.1, 136.6, 136.4, 129.6, 126.5, 125.6, 125.4, 121.7, 25.8, 18.1.

**6-methylquinoxaline (16b)**

Prepared according to GP1 from 6-bromoquinoxaline (52 mg, 0.25 mmol, 1.0 equiv.). Purification by flash column chromatography on silica gel (Heptane/EtOAc, 80:20) afforded the volatile product as colorless oil (33% yield, 12 mg). The spectroscopic data are in accordance with the literature.^[70]^

^1^H NMR (300 MHz, CDCl_3_) δ 8.79 (d, *J* = 7.5 Hz, 2H), 8.00 (d, *J* = 8.5 Hz, 1H), 7.88 (s, 1H), 7.62 (d, *J* = 8.5 Hz, 1H), 2.61 (s, 3H).

^13^C {1H} NMR (75 MHz, CDCl_3_) δ 145.1, 144.3, 143.3, 141.7, 140.8, 132.6, 129.2, 128.4, 21.0.

**1-methylisoquinoline (17b)**

Prepared according to GP1 from 1-bromoisoquinoline (52 mg, 0.25 mmol, 1.0 equiv.). Purification by flash column chromatography on silica gel (Pentane/EtOAc, pure pentane→9:1) afforded the volatile product as brown oil (31% yield, 11 mg). The spectroscopic data are in accordance with the literature.^[71]^

^1^H NMR (300 MHz, CDCl_3_) δ 8.39 (d, *J* = 5.8 Hz, 1H), 8.13 (d, *J* = 8.4 Hz, 1H), 7.81 (d, *J* = 7.7 Hz, 1H), 7.68 (ddd, *J* = 8.2, 6.8, 1.3 Hz, 1H), 7.60 (ddd, *J* = 8.3, 6.8, 1.4 Hz, 1H), 7.52 (d, *J* = 5.8 Hz, 1H), 2.97 (s, 3H).

^13^C {1H} NMR (75 MHz, CDCl_3_) δ 158.8, 141.8, 136.1, 130.1, 127.7, 127.4, 127.2, 125.8, 119.5, 22.5.

**Tert-butyl 5-methyl-1H-indole-1-carboxylate (18b)**

Prepared according to GP1 from tert-butyl 5-bromo-1H-indole-1-carboxylate (74 mg, 0.25 mmol, 1.0 equiv.). Purification by flash column chromatography on silica gel (Pentane/EtOAc, 99:1→97:2.5) afforded the product as white crystals (49% yield, 28 mg). The spectroscopic data are in accordance with the literature.^[72]^

^1^H NMR (300 MHz, CDCl_3_) δ 8.02 (d, *J* = 8.4 Hz, 1H), 7.56 (d, *J* = 3.7 Hz, 1H), 7.35 (s, 1H), 7.14 (d, *J* = 10.3 Hz, H), 6.50 (d, *J* = 3.7 Hz, 1H), 2.45 (s, 3H), 1.68 (s, 9H).

^13^C {1H} NMR (75 MHz, CDCl_3_) δ 150.0, 133.5, 132.2, 130.9, 126.0, 125.7, 121.0, 114.9, 107.1, 83.6, 28.4, 21.4.

**1,5-dimethyl-1H-benzo[*d*][1,2,3]triazole (19b)**

Prepared according to GP1 from 5-bromo-1-methyl-1H-benzo[d][1,2,3]triazole (53 mg, 0.25 mmol, 1.0 equiv.). Purification by flash column chromatography on silica gel (DCM/EtOAc, 99:1→95:5) afforded the product as brown solid (98% yield, 36 mg). The spectroscopic data are in accordance with the literature.^[73]^

^1^H NMR (300 MHz, CDCl_3_) δ 7.76 (s, 1H), 7.36 (d, *J* = 8.5 Hz, 1H), 7.28 (d, *J* = 8.5 Hz, 1H), 4.22 (s, 3H), 2.48 (s, 3H).

^13^C{1H} NMR (75 MHz, CDCl_3_) δ 146.6, 133.9, 132.1, 129.5, 118.8, 108.7, 34.3, 21.5.

**4-methyl-1-tosyl-1H-pyrrolo[2,3-*b*]pyridine (20b)**

Prepared according to GP1 from 4-bromo-1-tosyl-1H-pyrrolo[2,3-b]pyridine (88 mg, 0.25 mmol, 1.0 equiv.). Purification by flash column chromatography on silica gel (Pentane/EtOAc, 95:5→93:7) afforded the product as white solid (71% yield, 51 mg).

Preparation of **1b** according to GP2 from 4-bromo-1-tosyl-1H-pyrrolo[2,3-b]pyridine (352 mg, 1.0 mmol, 1.0 equiv.) afforded the desired product (84% yield, 240 mg).

^1^H NMR (300 MHz, CDCl_3_) δ 8.28 (d, *J* = 4.9 Hz, 1H), 8.04 (d, *J* = 8.4 Hz, 2H), 7.67 (d, *J* = 4.0 Hz, 1H), 7.23 (d, *J* = 8.2 Hz, 2H), 6.95 (d, *J* = 5.3 Hz, 1H), 6.60 (d, *J* = 4.0 Hz, 1H), 2.46 (s, 3H), 2.33 (s, 3H).

^13^C {1H} NMR (75 MHz, CDCl_3_) δ 147.1, 145.1, 144.9, 140.8, 135.5, 129.6, 127.9, 125.6, 123.0, 119.7, 103.8, 21.6, 18.2.

HRMS (FD) m/z calc’d for C_15_H_14_N_2_O_2_S: [M] 286.0776; found: 286.0780.

**5-methyl-1-tosylindoline (21b)**

Prepared according to GP1 from 5-bromo-1-tosylindoline (88 mg, 0.25 mmol, 1.0 equiv.). Purification by flash column chromatography on silica gel (Heptane/DCM, 60:40) afforded the product as colourless oil (40% yield, 29 mg). The spectroscopic data are in accordance with the literature.^[74]^

^1^H NMR (300 MHz, CDCl_3_) δ 7.65 (d, *J* = 8.3 Hz, 2H), 7.52 (d, *J* = 8.2 Hz, 1H), 7.21 (d, *J* = 7.9 Hz, 2H), 6.99 (d, *J* = 8.5 Hz, 1H), 6.88 (s, 1H), 3.88 (t, *J* = 8.3 Hz, 2H), 2.81 (t, *J* = 8.3 Hz, 2H), 2.36 (s, 3H), 2.26 (s, 3H).

^13^C {1H} NMR (75 MHz, CDCl_3_) δ 144.0, 139.8, 134.1, 133.6, 132.1, 129.7, 128.3, 127.5, 125.9, 115.1, 50.2, 28.0, 21.7, 21.0.

**methyl 6-methylnicotinate (22b)**

Prepared according to GP1 from methyl 6-bromonicotinate (54 mg, 0.25 mmol, 1.0 equiv.). Purification by flash column chromatography on silica gel (Pentane/EtOAc, 95:5pentane→90:10) afforded the product as yellow oil (56% yield, 21 mg). The spectroscopic data are in accordance with the literature.^[71]^

^1^H NMR (300 MHz, CDCl_3_) δ 9.07 (d, *J* = 1.5 Hz, 1H), 8.14 (dd, *J* = 8.1, 2.2 Hz, 1H), 7.21 (d, *J* = 8.1 Hz, 1H), 3.91 (s, 3H), 2.60 (s, 3H).

^13^C {1H} NMR (75 MHz, CDCl_3_) δ 166.1, 163.2, 150.6, 137.4, 123.4, 123.0, 52.3, 24.8.

**6-methyl-2,2'-bipyridine (23b)**

Prepared according to GP1 from 6-methyl-2,2'-bipyridine (59 mg, 0.25 mmol, 1.0 equiv.). Purification by flash column chromatography on silica gel (Pentane/EtOAc, 80:20) afforded the product as yellow oil (81% yield, 34 mg). The spectroscopic data are in accordance with the literature.^[71]^

^1^H NMR (300 MHz, CDCl_3_) δ 8.66 (ddd, *J* = 4.8, 1.9, 1.0 Hz, 1H), 8.40 (dt, *J* = 8.0, 1.1 Hz, 1H), 8.16 (d, *J* = 7.9 Hz, 1H), 7.79 (td, *J* = 7.7, 1.8 Hz, 1H), 7.69 (t, *J* = 7.7 Hz, 1H), 7.27 (ddd, *J* = 7.5, 4.8, 1.2 Hz, 1H), 7.16 (d, *J* = 7.6 Hz, 1H), 2.63 (s, 3H).

^13^C {1H} NMR (75 MHz, CDCl_3_) δ 158.1, 156.6, 155.7, 149.3, 137.2, 137.0, 123.6, 123.4, 121.3, 118.2, 24.8.

**5-methylpyrimidine-2-carbonitrile (24b)**

Prepared according to GP1 from 5-bromopyrimidine-2-carbonitrile (46 mg, 0.25 mmol, 1.0 equiv.). Purification by flash column chromatography on silica gel (Pentane/EtOAc, 90:10→75:25) afforded the product as yellow oil (80% yield, 24 mg). The spectroscopic data are in accordance with the literature.^[75]^

^1^H NMR (300 MHz, CDCl_3_) δ 8.67 (s, 2H), 2.43 (s, 3H).

^13^C {1H} NMR (75 MHz, CDCl_3_) δ 158.2, 142.9, 134.5, 115.9, 16.2.

**3-methyl-5-phenylpyridine (25b)**

Prepared according to GP1 from 3-bromo-5-phenylpyridine (59 mg, 0.25 mmol, 1.0 equiv.). Purification by flash column chromatography on silica gel (Pentane/EtOAc, 80:20) afforded the product as yellow oil (87% yield, 37 mg). The spectroscopic data are in accordance with the literature.^[76]^

^1^H NMR (300 MHz, CDCl_3_) δ 8.67 (s, 1H), 8.44 (s, 1H), 7.69 (s, 1H), 7.62 – 7.54 (m, 2H), 7.52 – 7.44 (m, 2H), 7.43 – 7.35 (m, 1H), 2.41 (s, 3H).

^13^C {1H} NMR (75 MHz, CDCl_3_) δ 149.0, 145.5, 138.0, 136.3, 135.2, 133.4, 129.2, 128.1, 127.3, 18.6.

**2-methyl-5-(methylsulfonyl)pyridine (26b)**

Prepared according to GP1 from 2-bromo-5-(methylsulfonyl)pyridine (59 mg, 0.25 mmol, 1.0 equiv.). Purification by flash column chromatography on silica gel (Pentane/EtOAc, 50:50→30:70) afforded the product as yellow flakes (89% yield, 38 mg).

^1^H NMR (300 MHz, CDCl_3_) δ 9.03 (d, *J* = 2.5 Hz, 1H), 8.10 (dd, *J* = 8.2, 2.4 Hz, 1H), 7.36 (d, *J* = 8.2 Hz, 1H), 3.09 (s, 3H), 2.68 (s, 3H).

^13^C {1H} NMR (75 MHz, CDCl_3_) δ 164.7, 148.3, 135.6, 134.4, 123.7, 45.1, 25.0.

HRMS (FD) m/z calc’d for C_7_H_9_N_1_O_2_S: [M] 171.0354; found: 171.0364.

**5-methyl-1-phenylpyridin-2(1H)-one (27b)**

Prepared according to GP1 from 5-bromo-1-phenylpyridin-2(1H)-one (63 mg, 0.25 mmol, 1.0 equiv.). Purification by flash column chromatography on silica gel (Heptane/EtOAc, 80:20) afforded the product as white solid (78% yield, 36 mg).

For the preparation of **27b** in continuous-flow a modified GP2 was adopted. A flame dried 50 mL round bottom flask was charged with the 5-bromo-1-phenylpyridin-2(1H)-one (1.0 equiv. 6.0 mmol, 1.50 g), DABAl-Me_3_ (1.25 equiv., 1.92 g, 7.50 mmol), NiCl_2_·dmg (5 mol%, 66 mg, 0.3 mmol), dtbpy (7.5 mol%, 121 mg, 0.45 mmol), 4CzIPN (5 mol%, 0.3 mmol, 237 mg) and evacuated and backfilled with N_2_. Subsequently, 2,6-lutidine (2.0 equiv., 2 mmol, 1.39 mL) and CH_3_CN (30 mL, dry and degassed via three cycles of freeze-pump-thaw) were added sequentially. The mixture was sonicated for 3 minutes and taken up by a 50 mL syringe. The syringe was mounted onto a syringe pump and connected to a PFA coil (V = 14.88 mL) which was placed into the Eagle reactor (λ_max_ = 450 nm). The reaction mixture was flowed through the reactor with a flow rate of 0.5 mL/min (30 min residence time) at 70% light intensity. When the syringe was fully empty, neat CH_3_CN (20 mL) was again loaded into a syringe and injected to collect all product at the end of the reactor in a flask. Evaporation and purification by flash column chromatography afforded the desired product (50% yield, 560 mg). The spectroscopic data are in accordance with the literature.^[52]^

^1^H NMR (300 MHz, CDCl_3_) δ = 7.52 – 7.38 (m, 2H), 7.43 – 7.31 (m, 3H), 7.30 – 7.18 (m, 1H), 7.10 (s, 1H), 6.59 (d, *J* = 9.3 Hz, 1H), 2.09 (s, 3H).

^13^C {1H} NMR (75 MHz, CDCl_3_) δ 161.9, 142.7, 141.2, 135.4, 129.4, 128.4, 126.6, 121.5, 114.9, 17.1.

**3-(benzyloxy)-5-methylpyridine (28b)**

Prepared according to GP1 from 3-(benzyloxy)-5-bromopyridine (66 mg, 0.25 mmol, 1.0 equiv.). Purification by flash column chromatography on silica gel (Heptane/EtOAc, 60:40) afforded the product as yellow oil (80% yield, 40 mg).

Preparation of **1b** according to GP2 from 4-bromo-1-tosyl-1H-pyrrolo[2,3-b]pyridine (264 mg, 1.0 mmol, 1.0 equiv.) afforded the desired product (75% yield, 150 mg).

^1^H NMR (300 MHz, CDCl_3_) δ 8.20 (d, *J* = 2.8 Hz, 1H), 8.07 (d, *J* = 1.7 Hz, 1H), 7.50 – 7.29 (m, 5H), 7.08 (s, 1H), 5.08 (s, 1H), 2.30 (d, *J* = 0.7 Hz, 1H).

^13^C {1H} NMR (75 MHz, CDCl_3_) δ 154.8, 143.0, 136.4, 135.3, 134.0, 128.8, 128.3, 127.6, 122.5, 70.3, 18.4.

HRMS (FD) m/z calc’d for C_13_H_13_N_1_O_1_: [M] 199.0997; found: 199.1009.

**2-(difluoromethoxy)-5-methylpyridine (29b)**

Prepared according to GP1 from 2-(difluoromethoxy)-5-bromopyridine (56 mg, 0.25 mmol, 1.0 equiv.). Purification by flash column chromatography on silica gel (Pentane/DCM, 90:10) afforded the volatile product as colourless oil (53% yield, 21 mg).^[53]^

^1^H NMR (300 MHz, CDCl_3_) δ 7.99 (d, *J* = 2.4 Hz, 1H), 7.52 (ddd, *J* = 8.4, 2.5, 0.7 Hz, 1H), 7.30 (t, *J* = 73.1 Hz, 1H), 6.80 (d, *J* = 8.3 Hz, 1H), 2.29 (s, 3H).

^13^C {1H} NMR (75 MHz, CDCl_3_) δ = 157.5 (t, *J* = 3.7 Hz,) 146.7, 140.9, 129.6, 114.4 (t, *J* = 254.7 Hz), 17.6.

^19^F NMR (282 MHz, CDCl_3_) δ -88.46 (d, *J* = 73.0 Hz).

**2-cyclobutoxy-6-methylpyridine (30b)**

Prepared according to GP1 2-bromo-6-cyclobutoxypyridine (57 mg, 0.25 mmol, 1.0 equiv.). Purification by flash column chromatography on silica gel (Pentane/Et_2_O, 98:2) afforded the volatile product as colorless oil (93% yield, 38 mg).

^1^H NMR (300 MHz, CDCl_3_) δ 7.43 (dd, *J* = 8.2, 7.2 Hz, 1H), 6.68 (d, *J* = 7.3 Hz, 1H), 6.45 (d, *J* = 8.2 Hz, H), 5.11 (ttd, *J* = 7.9, 7.0, 1.0 Hz, 1H), 2.55 – 2.36 (m, 5H), 2.24 – 2.01 (m, 2H), 1.83 (ddddd, *J* = 12.0, 9.8, 7.6, 2.6, 1.0 Hz, 1H), 1.77 – 1.58 (m, 1H).

^13^C {1H} NMR (75 MHz, CDCl_3_) δ 162.8, 156.7, 138.9, 115.8, 106.7, 69.9, 30.9, 24.4, 13.6.

HRMS (FD) m/z calc’d for C_10_H_13_N_1_O_1_: [M] 163.0997; found: 163.1016.

**5-methyl-2-(trifluoromethyl)pyrimidine (31b)**

Prepared according to GP1 from 5-bromo-2-(trifluoromethyl)pyrimidine (57 mg, 0.25 mmol, 1.0 equiv.). After the irradiation, 1-fluoronaphthalene (36.6 mg, 0.25 mmol, 1.0 equiv.) was added as internal standard and ^19^F-NMR analysis was performed, to confirm a yield of 95%.

^19^F NMR (282 MHz, CDCl_3_) δ -69.69.

HRMS (EI) m/z calc’d for C_6_H_4_F_3_N_2_: [M] 162.0405; found: 162.0397.

**2-methylbenzo[d]thiazole (32b)**

Prepared according to GP1 2-bromobenzo[d]thiazole (54 mg, 0.25 mmol, 1.0 equiv.). Purification by flash column chromatography on silica gel (Pentane/EtOAc, 95:5) afforded the volatile product as yellow oil (40% yield, 15 mg). The spectroscopic data are in accordance with the literature.^[77]^

^1^H NMR (300 MHz, CDCl_3_) δ 7.95 (d, *J* = 7.9 Hz, 1H), 7.82 (dd, *J* = 7.9, 0.7 Hz, 1H), 7.44 (ddd, *J* = 8.2, 7.2, 1.3 Hz, 1H), 7.38 – 7.30 (m, 1H), 2.84 (s, 3H).

^13^C {1H} NMR (75 MHz, CDCl_3_) δ 167.2, 153.4, 135.7, 126.1, 124.8, 122.5, 121.5, 20.2.

**2-methyldibenzo[*b*,*d*]furan (33b)**

Prepared according to GP1 2-bromodibenzo[b,d]furan (62 mg, 0.25 mmol, 1.0 equiv.). Purification by flash column chromatography on silica gel (Pentane/EtOAc, 98:2) afforded the product as colourless solid (83% yield, 38 mg). The spectroscopic data are in accordance with the literature.^[78]^

^1^H NMR (300 MHz, CDCl_3_) δ 7.93 (d, *J* = 7.6 Hz, 1H), 7.76 (s, 1H), 7.57 (d, *J* = 8.2 Hz, 1H), 7.50 – 7.41 (m, 2H), 7.37 – 7.24 (m, 2H), 2.53 (s, 3H).

^13^C {1H} NMR (75 MHz, CDCl_3_) δ 156.6, 154.7, 132.3, 128.3, 127.0, 124.4, 124.3, 122.6, 120.8, 120.7, 111.8, 111.3, 21.5.

**5-methylthianaphthene (34b)**

Prepared according toa modified GP1 from 5-bromobenzo[b]thiophene (53 mg, 0.25 mmol, 1.0 equiv.). The reaction was irradiated for 16 h. Purification by flash column chromatography on silica gel (Pentane/EtOAc, 100:0→99:1) afforded the product as yellow oil (71% yield, 26 mg). The spectroscopic data are in accordance with the literature.^[79]^

^1^H NMR (300 MHz, CDCl_3_) δ 7.78 (d, *J* = 8.2 Hz, 1H), 7.64 (s, 1H), 7.42 (d, *J* = 5.4 Hz, 1H), 7.28 (d, *J* = 6.2 Hz, 1H), 7.19 (d, *J* = 9.9 Hz, 1H), 2.50 (s, 3H).

^13^C {1H} NMR (101 MHz, CDCl_3_) δ 140.0, 136.9, 133.9, 126.4, 126.0, 123.6, 123.5, 122.12, 21.4.

**phenyl 5-methylthiophene-2-carboxylate (35b)**

Prepared according to GP1 from phenyl 5-bromothiophene-2-carboxylate (70.9 mg, 0.25 mmol, 1.0 equiv.). Purification by flash column chromatography on silica gel (Pentane/EtOAc, 75:25) afforded the product as white solid (83% yield, 45.3 mg).

^1^H NMR (300 MHz, CDCl_3_) δ 7.82 (d, *J* = 1.5 Hz, 1H), 7.49 – 7.40 (m, 2H), 7.33 – 7.20 (m, 4H), 2.35 (s, 3H).

^13^C {1H} NMR (101 MHz, CDCl_3_) δ 160.7, 150.7, 138.8, 136.6, 132.4, 129.5, 129.4, 126.0, 121.7, 15.6.

HRMS (FD) m/z calc’d for C_13_H_13_N_1_O_1_: [M] 218.0401; found: 218.0394.

# 8. References

[46]	L. D. Haire, P. H. Krygsman, E. G. Janzen, U. M. Oehler, *J. Org. Chem.* **1988**, *53*, 4535–4542.

[50]	N. Alandini, L. Buzzetti, G. Favi, T. Schulte, L. Candish, K. D. Collins, P. Melchiorre, *Angew. Chem. Int. Ed.* **2020**, *59*, 5248–5253.

[51]	A. Mahindra, O. Janha, K. Mapesa, A. Sanchez-Azqueta, M. M. Alam, A. Amambua-Ngwa, D. C. Nwakanma, A. B. Tobin, A. G. Jamieson, *J. Med. Chem.* **2020**, *63*, 9300–9315.

[52]	E. Falb, K. Ulanenko, A. Tor, R. Gottesfeld, M. Weitman, M. Afri, H. Gottlieb, A. Hassner, *Green Chem.* **2017**, *19*, 5046–5053.

[53]	Z. Zhu, V. Krishnamurti, X. Ispizua-Rodriguez, C. Barrett, G. K. S. Prakash, *Org. Lett.* **2021**, *23*, 6494–6498.

[54]	Y. Wu, Y.-H. Huang, X.-Y. Chen, P. Wang, *Org. Lett.* **2020**, *22*, 6657–6661.

[55]	S. Stoll, A. Schweiger, *J. Magn. Reson.* **2006**, *178*, 42–55.

[56]	Thomas Casey (2024). cwEPR (https://nl.mathworks.com/matlabcentral/fileexchange/73292-cwepr), MATLAB Central File Exchange.

[57]	R. D. Bradley, B. D. McManus, J. G. Yam, V. Carta, A. Bahamonde, *Angew. Chem. Int. Ed.* **2023**, e202310753.

[58]	J. Cheng, J. Xie, Y. Xi, X. Wu, R. Zhang, Z. Mao, H. Yang, Z. Li, C. Li, *Angew. Chem. Int. Ed.* **2024**, e202319896.

[59]	V. W. Bhoyare, A. Bera, V. Gandon, N. T. Patil, *Angew. Chem. Int. Ed.* **2024**, e202410794.

[60]	R. Sun, G. Li, F. Xie, Q. Zhang, Y.-J. Sun, W. Dai, *Org. Lett.* **2024**, *26*, 928–932.

[61]	J. Yang, Y. Wu, X. Wu, W. Liu, Y. Wang, J. Wang, *Green Chemistry* **2019**, *21*, 5267–5273.

[62]	A. Cook, S. Prakash, Y.-L. Zheng, S. G. Newman, *J. Am. Chem. Soc.* **2020**, *142*, 8109–8115.

[63]	K. Yang, J. Lu, L. Li, S. Luo, N. Fu, *Chem. Eur. J.* **2022**, e202202370.

[64]	C. C. D. Wybon, C. Mensch, C. Hollanders, C. Gadais, W. A. Herrebout, S. Ballet, B. U. W. Maes, *ACS Catal.* **2018**, *8*, 203–218.

[65]	H. Wang, W. Han, A. Noble, V. K. Aggarwal, *Angew. Chem. Int. Ed.* **2022**, e202207988.

[66]	T. Nishikata, H. Nagashima, *Angew. Chem. Int. Ed.* **2012**, *51*, 5363–5366.

[67]	P. Novák, A. Lishchynskyi, V. V. Grushin, *Angew. Chem. Int. Ed.* **2012**, *51*, 7767–7770.

[68]	Q. Wu, S. Han, X. Ren, H. Lu, J. Li, D. Zou, Y. Wu, Y. Wu, *Org. Lett.* **2018**, *20*, 6345–6348.

[69]	T. J. Stumpf, M. Steinbach, M. Höltke, G. Heuger, F. Grasemann, R. Fröhlich, S. Schindler, R. Göttlich, Eur. *J. Org. Chem.* **2018**, *2018*, 5538–5547.

[70]	A. Yu. Dubovtsev, N. V. Shcherbakov, D. V. Dar’in, V. Yu. Kukushkin, *J. Org. Chem.* **2020**, *85*, 745–757.

[71]	S. Han, P. Chakrasali, J. Park, H. Oh, S. Kim, K. Kim, A. K. Pandey, S. H. Han, S. B. Han, I. S. Kim, *Angew. Chem. Int. Ed.* **2018**, *57*, 12737–12740.

[72]	S. Pandit, V. K. Pandey, A. S. Adhikari, S. Kumar, A. K. Maurya, R. Kant, N. Majumdar, *J. Org. Chem.* **2023**, *88*, 97–105.

[73]	F. Ospina, K. H. Schülke, J. Soler, A. Klein, B. Prosenc, M. Garcia‐Borràs, S. C. Hammer, *Angew. Chem. Int. Ed.* **2022**, e202213056.

[74]	A. Padwa, M. A. Brodney, B. Liu, K. Satake, T. Wu, *J. Org. Chem.* **1999**, *64*, 3595–3607.

[75]	P. Adhikari, G. Li, M. Go, D. Mandikian, H. Rafidi, C. Ng, S. Anifa, K. Johnson, L. Bao, H. Hernandez Barry, R. Rowntree, N. Agard, C. Wu, K.-J. Chou, D. Zhang, K. R. Kozak, T. H. Pillow, G. D. Lewis, S.-F. Yu, C. A. Boswell, J. D. Sadowsky, *J. Am. Chem. Soc.* **2024**, *146*, 19088–19100.

[76]	C.-H. Hu, Y. Sang, Y.-W. Yang, W.-W. Li, H.-L. Wang, Z. Zhang, C. Ye, L.-Z. Wu, X.-S. Xue, Y. Li, *Chem* **2023**, *9*, 2997–3012.

[77]	M. Tomar, R. Bhimpuria, D. Kocsi, A. Thapper, K. E. Borbas, *J. Am. Chem. Soc.* **2023**, *145*, 22555–22562.

[78]	G. J. Clarkson, S. Roesner, *J. Org. Chem.* **2023**, *88*, 684–689.

[79]	Y. Peng, M. Oestreich *Org. Lett.* **2022**, *24*, 2940–2943.

# 9. NMR spectra

Compound **7a:** ^1^H-NMR spectrum (300 MHz, CDCl_3_)

Compound **7a:** ^13^C {1H} NMR (75 MHz, CDCl_3_)

Compound **8a:** ^1^H-NMR spectrum (300 MHz, CDCl_3_)

Compound **8a:** ^13^C {1H} NMR (75 MHz, CDCl_3_)

Compound **21a:** ^1^H-NMR spectrum (300 MHz, CDCl_3_)

Compound **21a:** ^13^C {1H} NMR (75 MHz, CDCl_3_)

Compound **30a:** ^1^H-NMR spectrum (300 MHz, CDCl_3_)

Compound **30a:** ^13^C {1H} NMR (75 MHz, CDCl_3_)

Compound **35a:** ^1^H-NMR spectrum (300 MHz, CDCl_3_)

Compound **35a:** ^13^C {1H} NMR (101 MHz, CDCl_3_)

Compound **39:** ^1^H-NMR spectrum (300 MHz, C_6_D_6_)

Compound **39:** ^19^F-NMR spectrum (282 MHz, C_6_D_6_)

Compound **1b:** ^1^H-NMR spectrum (300 MHz, CDCl_3_)

Compound **1b:** ^13^C {1H} NMR (75 MHz, CDCl_3_)

Compound **2b:** ^1^H-NMR spectrum (300 MHz, CDCl_3_)

Compound **2b:** ^13^C {1H} NMR (75 MHz, CDCl_3_)

Compound **3b:** ^1^H-NMR spectrum (300 MHz, CDCl_3_)

Compound **3b:** ^13^C {1H} NMR (75 MHz, CDCl_3_)

Compound **4b:** ^1^H-NMR spectrum (300 MHz, CDCl_3_)

Compound **4b:** ^13^C {1H} NMR (101 MHz, CDCl_3_)

Compound **5b:** ^1^H-NMR spectrum (300 MHz, CDCl_3_)

Compound **5b:** ^13^C {1H} NMR (101 MHz, CDCl_3_)

Compound **6b:** ^1^H-NMR spectrum (300 MHz, CDCl_3_)

Compound **6b:** ^13^C {1H} NMR (101 MHz, CDCl_3_)

Compound **7b:** ^1^H-NMR spectrum (300 MHz, CDCl_3_) Compound **7b:** ^13^C {1H} NMR (75 MHz, CDCl_3_) Compound **8b:** ^1^H-NMR spectrum (300 MHz, CDCl_3_) Compound **8b:** ^13^C {1H} NMR (75 MHz, CDCl_3_)

Compound **9b:** ^1^H-NMR spectrum (300 MHz, CDCl_3_)

Compound **9b:** ^1^H-NMR spectrum (150 MHz, CDCl_3_)

Compound **10b** ^19^F-NMR spectrum (282 MHz, CDCl_3_)

Compound **11b:** ^1^H-NMR spectrum (300 MHz, CDCl_3_) Compound **11b:** ^13^C {1H} NMR (75 MHz, CDCl_3_)

Compound **12b** ^1^H-NMR spectrum (300 MHz, CDCl_3_)

Compound **12b:** ^13^C {1H} NMR (75 MHz, CDCl_3_)

Compound **13b:** ^1^H-NMR spectrum (300 MHz, CDCl_3_)

Compound **13b:** ^13^C-NMR spectrum (75 MHz, CDCl_3_)

Compound **14b:** ^1^H-NMR spectrum (300 MHz, CDCl_3_)

Compound **14b:** ^13^C {1H} NMR (75 MHz, CDCl_3_)

Compound **15b:** ^1^H-NMR spectrum (300 MHz, CDCl_3_)Compound **15b:** ^13^C-NMR spectrum (75 MHz, CDCl_3_)

Compound **16b:** ^1^H-NMR spectrum (300 MHz, CDCl_3_)

Compound **16b:** ^13^C {1H} NMR (75 MHz, CDCl_3_)

Compound **17b:** ^1^H-NMR spectrum (300 MHz, CDCl_3_)

Compound **17b:** ^13^C {1H} NMR (75 MHz, CDCl_3_)

Compound **18b:** ^1^H-NMR spectrum (300 MHz, CDCl_3_)

Compound **18b:** ^13^C {1H} NMR (75 MHz, CDCl_3_)

Compound **19b:** ^1^H-NMR spectrum (300 MHz, CDCl_3_)

Compound **19b:** ^13^C {1H} NMR (75 MHz, CDCl_3_)

Compound **20b:** ^1^H-NMR spectrum (300 MHz, CDCl_3_)

Compound **20b:** ^13^C {1H} NMR (75 MHz, CDCl_3_)

Compound **21b:** ^1^H-NMR spectrum (300 MHz, CDCl_3_)

Compound **21b:** ^13^C {1H} NMR (75 MHz, CDCl_3_)

Compound **22b:** ^1^H-NMR spectrum (300 MHz, CDCl_3_)

Compound **22b:** ^13^C {1H} NMR (75 MHz, CDCl_3_)

Compound **23b:** ^1^H-NMR spectrum (300 MHz, CDCl_3_)

Compound **23b:** ^13^C {1H} NMR (75 MHz, CDCl_3_)

Compound **24b:** ^1^H-NMR spectrum (300 MHz, CDCl_3_)

Compound **24b:** ^13^C {1H} NMR (75 MHz, CDCl_3_)

Compound **25b:** ^1^H-NMR spectrum (300 MHz, CDCl_3_)

Compound **25b:** ^13^C {1H} NMR (75 MHz, CDCl_3_)

Compound **26b:** ^1^H-NMR spectrum (300 MHz, CDCl_3_)

Compound **26b:** ^13^C {1H} NMR (75 MHz, CDCl_3_)

Compound **27b:** ^1^H-NMR spectrum (300 MHz, CDCl_3_)

Compound **27b:** ^13^C {1H} NMR (75 MHz, CDCl_3_)

Compound **28b:** ^1^H-NMR spectrum (300 MHz, CDCl_3_)

Compound **28b:** ^13^C {1H} NMR (75 MHz, CDCl_3_)

Compound **29b:** ^1^H-NMR spectrum (300 MHz, CDCl_3_)

Compound **29b:** ^13^C {1H} NMR (75 MHz, CDCl_3_)

Compound **29b:** ^19^F NMR (282 MHz, CDCl_3_)

Compound **30b:** ^1^H-NMR spectrum (300 MHz, CDCl_3_)

Compound **30b:** ^13^C {1H} NMR (75 MHz, CDCl_3_)

Compound **31b:** ^19^F NMR (282 MHz, CDCl_3_)

Compound **32b:** ^1^H-NMR spectrum (300 MHz, CDCl_3_)

Compound **32b:** ^13^C {1H} NMR (75 MHz, CDCl_3_)

Compound **33b:** ^1^H-NMR spectrum (300 MHz, CDCl_3_)

Compound **33b:** ^13^C {1H} NMR (75 MHz, CDCl_3_)

Compound **34b:** ^1^H-NMR spectrum (300 MHz, CDCl_3_)

Compound **34b:** ^13^C {1H} NMR (75 MHz, CDCl_3_)

Compound **35b:** ^1^H-NMR spectrum (300 MHz, CDCl_3_)

 Compound **35b:** ^13^C {1H} NMR (101 MHz, CDCl_3_)
